# Effects of wildfire on soil microbial communities in karst forest ecosystems of southern Guizhou Province, China

**DOI:** 10.1128/aem.01245-24

**Published:** 2024-10-30

**Authors:** Xu Li, Yanfeng Han, Yunlin Zhang, Qiuyu Shao, Chunbo Dong, Jianfeng Li, Bo Ding, Yanwei Zhang

**Affiliations:** 1 Department of Ecology/Key Laboratory of Plant Resource Conservation and Germplasm Innovation in Mountainous Region (Ministry of Education), Institute of Fungus Resources, College of Life Sciences, Guizhou University, Guiyang, Guizhou, China; 2 Key Laboratory of Ecology and Management on Forest Fire in Higher Education institutions of Guizhou Province/Key Laboratory of Development and Utilization of Biological Resources in Colleges and Universities of Guizhou Province, Guizhou Education University, Guiyang, Guizhou, China; Colorado School of Mines, Golden, Colorado, USA

**Keywords:** wildfire, fungal community, bacterial community, soil physicochemical factors, Mantel test

## Abstract

Wildfires are unpredictable disturbances with profound effects on soil properties and microbial communities within forest ecosystems. However, knowledge of post-fire microbial communities in karst forests remains limited. In this study, microbial amplicon sequencing techniques were employed to investigate the impact of wildfires on the composition, diversity, function, and co-occurrence network of soil microbial communities in karst forest landscapes and to identify the key soil physicochemical factors affecting the post-fire microbial communities. The wildfire affected the fungal community to a greater extent than the bacterial community, with the former shifting from a dominance of Basidiomycota to Ascomycota at the phylum level, while the relative abundance of Actinobacteria increased significantly in the bacterial community. Moreover, the wildfire increased the α-diversity of the microbial community and changed the β-diversity. Network analysis indicated significant reductions in the complexity of microbial community networks and the hub microbiome in burned soils compared to those of unburned soils. Functional predictions indicated an increase in the highly abundant functional taxa of chemoheterotrophic and aerobic chemoheterotrophic bacteria, along with a significant rise in saprotrophic functional fungal taxa following the fire. In addition, soil organic matter, total nitrogen, total phosphorus, and soil water content emerged as key soil physicochemical factors affecting post-fire soil microbial communities in the karst forest. Overall, this study revealed the structural and functional characteristics of soil microbial communities and their key influencing factors after a fire in a karst forest, which will provide a valuable theoretical basis for ecosystem restoration after a wildfire.

**IMPORTANCE:**

Despite the significant impacts of wildfires on forest ecosystems, most existing studies have largely focused on boreal and Mediterranean coniferous forest types, with limited research on the impacts of coniferous and broadleaf forest types in subtropical karst regions. This study reveals the effects of wildfires on soil microbial communities of coniferous and broadleaf forest types in a karst forest. The results of this study not only improve the understanding of the effects of wildfires on the composition, diversity, function, and network of soil microbial communities but also provide a meaningful theoretical basis for post-fire ecosystem restoration in the karst forest.

## INTRODUCTION

The increasing warming of the global climate and extreme weather conditions have led to an increased frequency of wildfires. From 2000 to 2021, China experienced an average of 5,688 wildfires per year, resulting in the burning of up to 73,964 ha of forest (http://www.stats.gov.cn/tjsj/ndsj/) (1), making it one of the countries with the most serious forest fires. Wildfires not only destroy valuable forest resources but also cause severe disturbance to the microbial communities of forest ecosystems (2). These communities play important roles in nutrient cycling, energy flow, and information transfer and thus have a substantial functional impact on the recovery process after wildfires (3–5). Therefore, understanding how wildfires affect the soil microbial communities in forest ecosystems is of great significance for post-fire ecosystem restoration.

In general, the effects of forest fires on soil microbial communities are categorized into direct and indirect effects. Direct effects include cell death due to high-temperature stress, leading to a reduction in microbial relative abundance and diversity (6, 7), whereas indirect effects mainly involve changes in soil physicochemical properties, nutrients, and the microenvironment after fire. These indirect effects impact microbial communities mainly through changes in soil organic matter (SOM), soil water content (SWC), soil pH, and extracellular enzyme activity, as well as the accessible nutrient supply (8, 9). All of these effects have implications for carbon and nitrogen cycling, mineral nutrient transformations, and other processes that regulate the forest ecosystem during restoration (10). During succession of forest ecosystems after a fire, the direct effects of the fire on microbial communities may become less important, while the indirect effects exert a major influence on microbial communities and play a positive role in the recovery of post-fire forests (11).

Microorganisms not only are involved in nutrient cycling and organic matter transformation in the soil but also modify the soil environment; this microbial-mediated modification of soil properties can in turn have an impact on the composition of the microbial community and thus has important ecosystem implications. There is a close interaction between microbial communities and soil properties (12). Fire leads to the burning of numerous organic materials such as vegetation and litter, thereby increasing available nutrients and altering soil physicochemical properties (13, 14), ultimately causing changes in soil microbial communities. Previous studies showed that post-fire pyrogenic organic matter (PyOM) formation results in significant changes in microbial compositional abundance, with a significant enrichment in Actinobacteria and a significant decline in Ascomycetes abundance in the post-fire soil (15, 16). In addition, post-fire changes in soil pH result in significant changes in microbial diversity and were identified as key determinants in post-fire microbial recovery (17). With respect to functional impacts, Sun et al. (18) showed that the relative contribution of ectomycorrhizal fungi to the community increased during post-fire recovery, which was mainly driven by the available SOM after the fire. Furthermore, post-fire soil microbial networks and the hub microbiome were found to be affected by the changes in SWC, pH, and nitrogenous compounds occurring during post-fire recovery (13, 14, 19).

Despite numerous studies on the interactions between soil microbial communities and soil physicochemical factors after fire, these studies have mainly focused on ecosystems of coniferous forest types in boreal and Mediterranean regions, whereas short-term post-fire recovery studies for tropical and subtropical ecosystems of both coniferous and broadleaf forest types remain limited (13, 19). In particular, such studies are notably lacking for karst forest areas.

The karst forest ecosystems in southern China have unique geomorphic structures, characterized by high habitat heterogeneity, shallow and discontinuous soils, multilayered ecological space, and extreme fragility; however, these forests play an important role in preventing soil erosion (20). Guizhou Province exhibits a typical karst landscape development with a widespread distribution area. Between 2001 and 2020, a total of 15,754 fires were recorded in the province, covering an area exceeding 175,000 hm^2^. These fires have had a significant impact on the forest ecosystem, resulting in substantial direct and indirect economic losses. Therefore, determining the best strategy to quickly renew and restore forest land after a fire is not only an important ecological issue but also an important issue for sustainable development.

In this study, we used 16S rRNA and internal transcribed spacer (ITS) gene amplicon sequencing to investigate the characteristics of soil microbial communities in burned and unburned coniferous and broadleaf forests in karst regions of China, and we further explored the impact of the post-fire soil physicochemical properties on microbial community construction. The aims of this study were therefore to (1) evaluate the immediate impact of forest fires on the composition, diversity, function, and symbiotic network of soil bacterial and fungal communities in karst forests (2); compare the similarities and differences in soil bacterial and fungal communities between coniferous and broadleaf forests following fire; and (3) identify the key soil physicochemical factors influencing the soil microbial communities in karst forests after fires. These findings will provide information for understanding the short-term recovery of soil microbial communities in burned areas of karst forest ecosystems.

## RESULTS

### Composition and diversity of soil microorganisms in burned and unburned forests

Deep sequencing of the 16S rRNA gene V3–V4 region yielded 1,563,999 raw reads from 20 samples, resulting in 1,027,687 high-quality reads (range: 32,792–62,131) and 41,621 amplicon sequence variants (ASVs) (range: 1,705–3,123) (Table S2). Deep sequencing of the ITS1 region generated a total of 1,599,851 raw reads for 20 samples, obtaining 1,274,257 high-quality reads (range: 41,363–69,817), forming 10,235 ASVs (range: 487–862) (Table S2).

**Fig 1:**
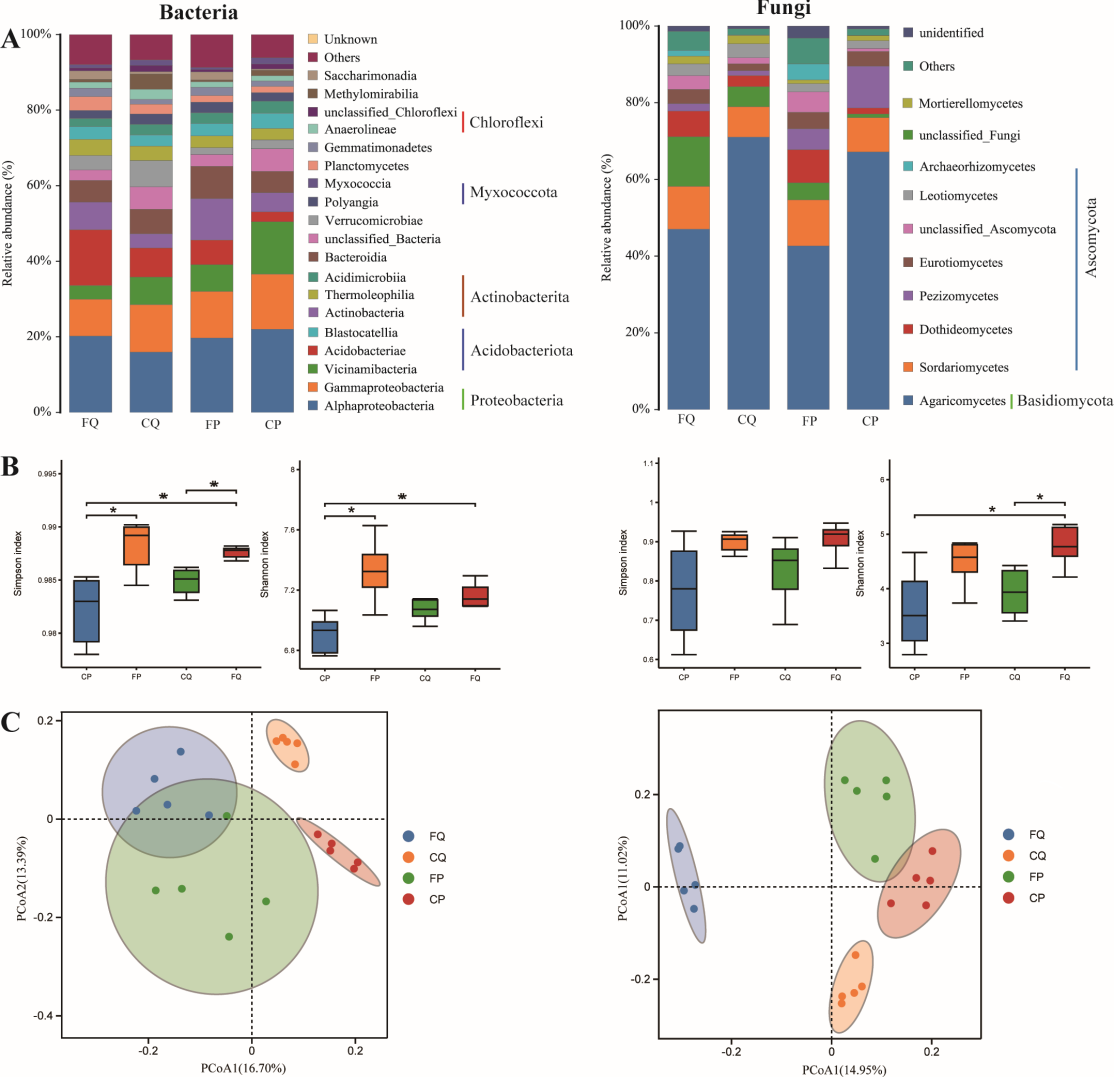
Composition and diversity of soil microbial community in burned and unburned forests. (**A**) Difference in the composition of microbial community between burned and unburned. Low abundance classes with a total sequence number of less than 1% in all samples were classified as “Other.” (**B**) Differences in α-diversity of microbial community between burned and unburned were determined using a nonparametric Wilcoxon rank-sum test, with *P* < 0.05 indicating significant difference. *Indicates significance, with significance levels of *P  <  0.05 and * *P  <  0.01. (**C**) β-Diversity of microbial community between burned and unburned. Principal coordinate analysis at the ASV level based on binary-jaccard distances. Abbreviations used are as follows: unburned *Quercus fabri* forest (CQ), burned *Quercus fabri* forest (FQ), unburned *Pinus massoniana* forest (CP), and burned *Pinus massoniana* forest (FP).

In terms of microbial composition, Alphaproteobacteria and Agaricomycetes were dominant (the relative abundance is >15%) among burned *Quercus fabri* forest (FQ), unburned *Quercus fabri* forest (CQ), burned *Pinus massoniana* forest (FP), and unburned *Pinus massoniana* forest (CP). The soil bacteria at all sites were mainly composed of Alphaproteobacteria, Gammaproteobacteria, Vicinamibacteria, and Acidobacteriae, and the fungi were mainly composed of Agaricomycetes. In both the coniferous and broadleaf forests, fire caused a decrease in the relative abundances of Gammaproteobacteria and Vicinamibacteria, with a decrease of 2.28% and 6.81%, respectively, in the coniferous forest. Fire also significantly elevated the relative abundance of certain bacterial taxa. The relative abundance of Actinobacteria increased from 5.07% in unburned coniferous forests to 10.96% after the fire, while the relative abundance of Acidobacteriota increased from 7.69% to 14.70% after the fire in unburned broadleaf forests (Table S3). In addition, the impact of fire on the relative abundance of Alphaproteobacteria differed between coniferous and broadleaf forest types. In the coniferous forest, the relative abundance decreased from 22.62% in unburned areas to 19.73% after the fire. Conversely, in broadleaf forests, the relative abundance of Alphaproteobacteria increased by 4.26% following the fire. For the fungal community, we also found a notable reduction in the relative abundance of Agaricomycetes at the class level following exposure to fire, with a decrease from 71.01% to 46.44% in the broadleaf forest and a decrease from 66.95% to 41.40% in the coniferous forest (Fig. 1A; Table S3). Fire increased the relative abundance of classes related to Ascomycota, with the exception of Leotiomycetes in the broadleaf forest and Pezizomycetes in the coniferous forest.

Fire increased soil microbial community diversity in both the broadleaf and coniferous forests. The Simpson index of the bacterial community was significantly different between burned and unburned forests (*P* < 0.05), whereas there was no significant difference in the Simpson index of the fungal community (Fig. 1B). The Shannon index of the bacterial community was significantly different between burned and unburned coniferous forests (*P* < 0.05). By contrast, the Shannon index of the fungal community did not significantly differ between burned and unburned coniferous forests but had a significant difference (*P* < 0.05) between burned and unburned broadleaf forests (Fig. 1B).

Principal coordinate analysis (PCoA) based on the binary-Jaccard distance revealed differences in bacterial and fungal community structures between burned and unburned forests. The bacterial and fungal communities were clearly separated between burned and unburned broadleaf and coniferous forests, and bacterial communities were relatively more dispersed in the burned forests than in the unburned forests (Fig. 1C). Permutational multivariate analysis of variance (PERMANOVA) showed that fungal communities (broadleaf forests: R^2^ = 0.288, *P* = 0.001; coniferous forests: R^2^ = 0.192, *P* = 0.001) were more affected than bacterial communities (broadleaf forests: R^2^ = 0.273, *P* = 0.001; coniferous forests: R^2^ = 0.025, *P* = 0.001) after fire in both forest types. We also found significant (*P* = 0.001) dissimilarity in bacterial and fungal community structures among study sites (Table S4).

### Co-occurrence networks of soil microorganisms in burned and unburned forests

**Fig 2:**
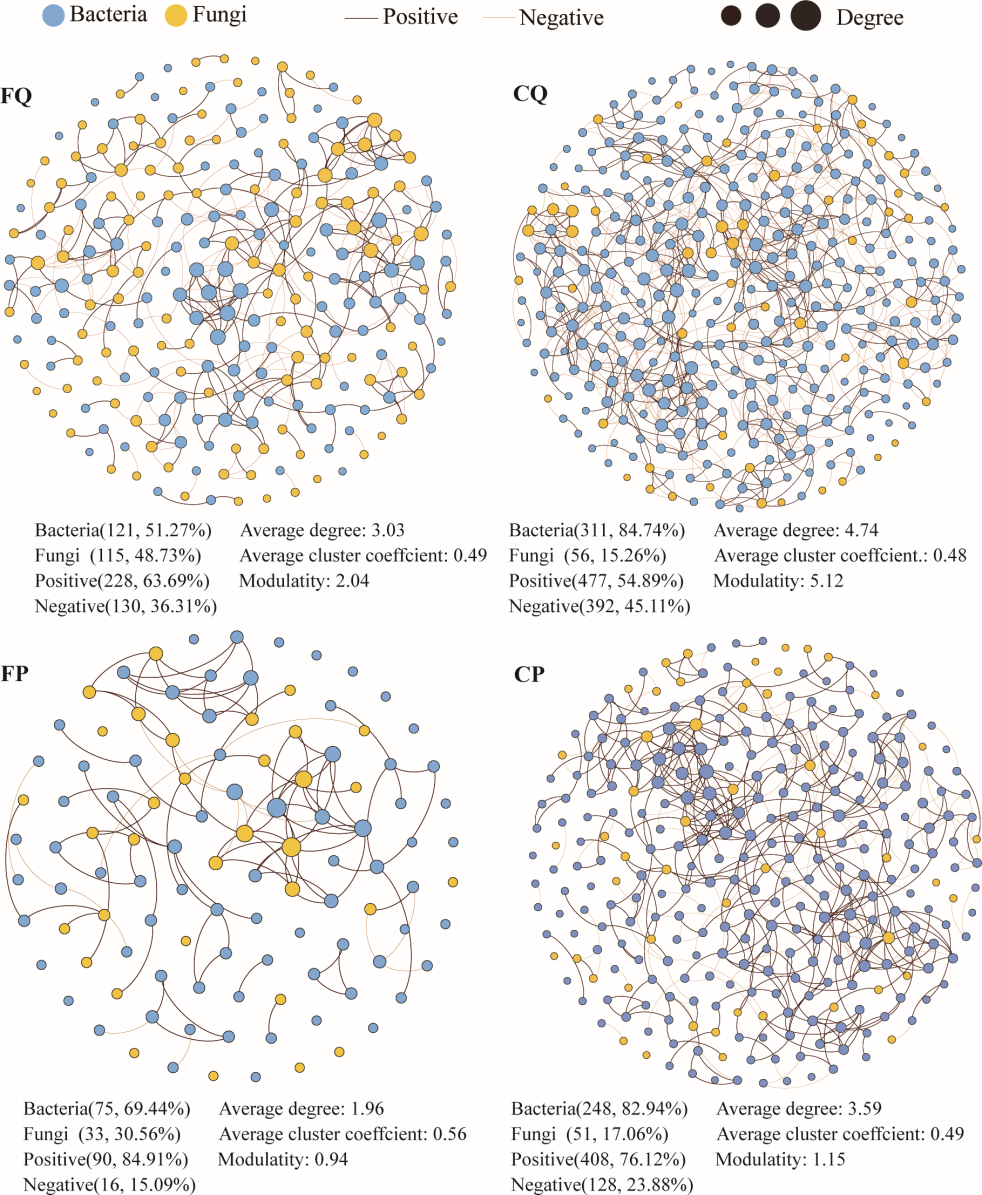
Co-occurrence networks of soil microbial community (ASV levels) in burned and unburned forests. Abbreviations used are as follows: unburned *Quercus fabri* forest (CQ), burned *Quercus fabri* forest (FQ), unburned *Pinus massoniana* forest (CP), and burned *Pinus massoniana* forest (FP).

Network analysis revealed differences in microbial co-occurrence patterns between burned and unburned soils in broadleaf and coniferous forests (Fig. 2). The results showed that both the burned and unburned microbial co-occurrence networks were mainly positively correlated, although the microbial community in the burned forests was simpler than that identified in the corresponding unburned state. For example, in the broadleaf forest, the network that formed after the fire (FQ) consisted of 236 nodes and 358 edges, while the unburned network (CQ) consisted of 367 nodes and 869 edges. In the coniferous forest, the network formed after the fire (FP) consisted of 106 nodes and 108 edges, while the unburned network (CP) consisted of 299 nodes and 536 edges. Compared to the unburned state, the average degree of network connectivity and module number decreased while the average clustering coefficient increased. For instance, in the broadleaf forest, the average network connectivity decreased from 4.74 to 3.03 and the number of modules decreased from 5.12 to 2.04, while the average clustering coefficient increased from 0.48 to 0.49. In the coniferous forest, the average network connectivity decreased from 3.59 to 1.96 and the number of modules decreased from 1.15 to 0.94, while the average clustering coefficient increased from 0.49 to 0.56.

We further defined the “hub microbiome” as nodes with high degree (≥7) and high closeness centrally (≥0.08) in the network. In the broadleaf forests, there were 91 ASVs identified in the unburned *Q. fabri* forests, which decreased to 24 after burning. These hub microbiomes included the genera *Roseiarcus*, *Mycobacterium*, *Occallatibacter*, *Methylobacterium Methylorubrum*, *Bryobacter*, *Chloridium*, *Phialocephala*, *Oidiodendron*, *Articulospora*, *Udeniomyces*, and 14 undefined taxa (Table S5). In the coniferous forest, there were 36 ASVs identified in the unburned *P. massoniana* forests, which decreased to five after burning. These hub microbiomes included the genera *Aridibacter*, *Flavisolibacter*, *Solicoccozyma*, *Gyrothrix*, and *Aspergillus* (Table S6). The results also showed that the proportion of bacteria nodes decreased after the fire in both broadleaf and coniferous forests, whereas the proportion of fungi nodes increased after burning.

### Functional characteristics of soil microorganisms in burned and unburned forests

**Fig 3:**
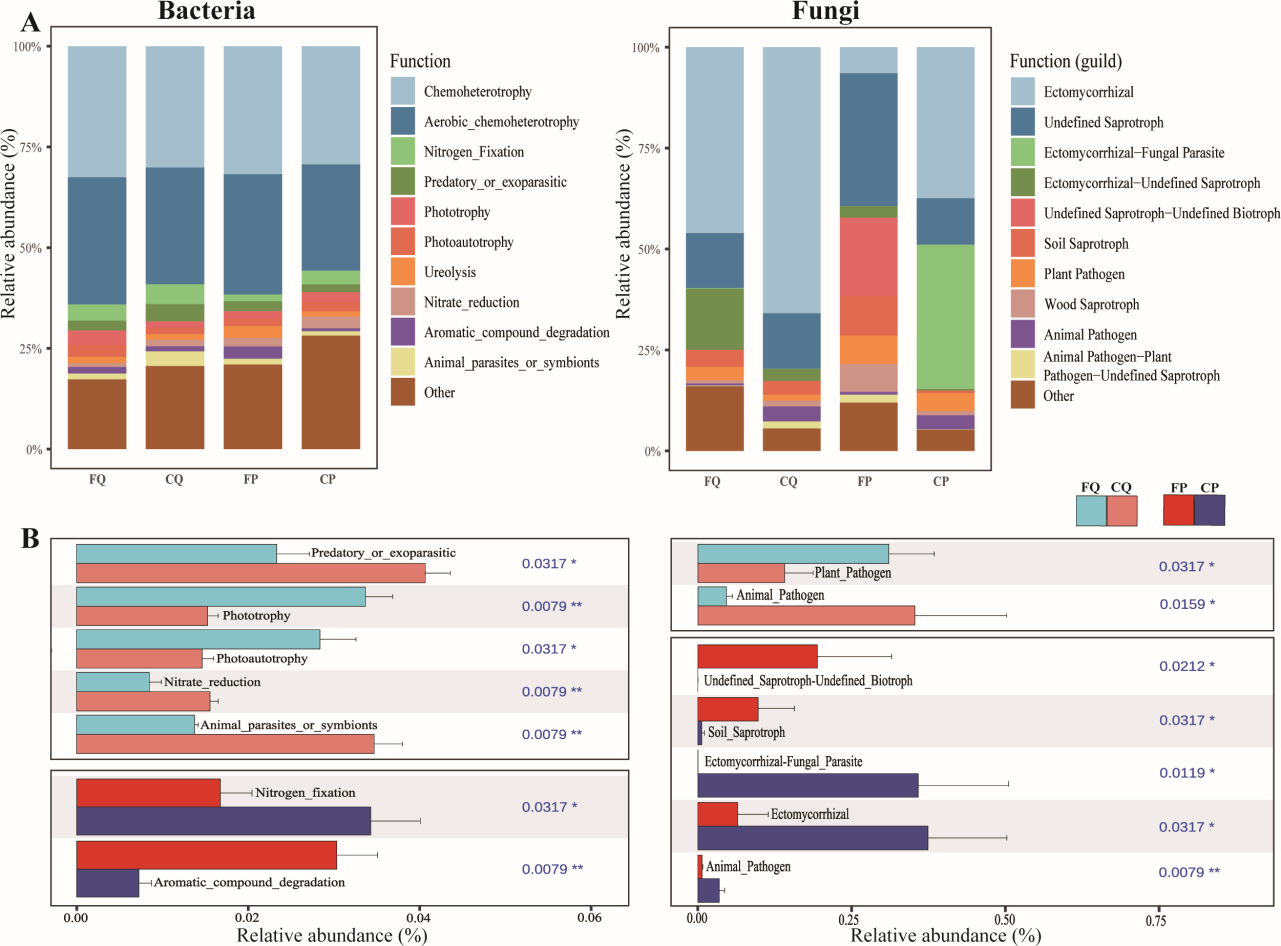
Functional characteristics of soil microbial community in burned and unburned forests. (**A**) Functional (top 10 in abundance) composition of bacterial and fungal communities in burned and unburned. (**B**) Differences in bacterial and fungal community functions (top 10 in abundance) between burned and unburned were determined using nonparametric Wilcoxon rank-sum test (10 pairs of functional characteristics). Figure shows only the functional groups with significant (*P* < 0.05) levels of difference in abundance between burned and unburned samples. Significance levels were *P < 0.05, **P < 0.01, and ***P < 0.001.

Burned and unburned bacterial communities were annotated into 53 functional groups. These functional types exhibited abundance differences between burned and unburned samples, such as an increase in the relative abundance of functional groups of chemoheterotrophic and aerobic chemoheterotrophic bacteria after fire (Fig. 3A). In the burned broadleaf forest, the relative abundances of chemoheterotrophic and aerobic chemoheterotrophic bacteria were 32.53% and 31.54%, respectively, while their relative abundances in the unburned broadleaf forest were 30.11% and 29%, respectively. In the coniferous forest, the relative abundances of chemoheterotrophic and aerobic chemoheterotrophic bacteria in the burned state were 31.77% and 29.87%, respectively, while their relative abundances in the unburned state were 29.34% and 26.36%, respectively. The Wilcoxon rank-sum test further confirmed the differences between burned and unburned bacterial community functions (Fig. 3B). In broadleaf forests, the relative abundances of bacteria with functions annotated as predatory or exoparasitic, nitrate reduction, and animal parasites or symbionts were significantly decreased after the fire. Conversely, the relative abundances of bacteria with functions of phototrophy and photoautotrophy were significantly higher after the fire (Table S7). In the coniferous forest, the relative abundance of bacteria with a nitrogen-fixation function significantly decreased after burning, while the relative abundance of bacteria with a function of aromatic compound degradation significantly increased after the fire (Table S8). The relative abundances of bacteria with functions of phototrophy, photoautotrophy, and nitrogen fixation were significantly higher in the broadleaf forest than in the coniferous forest after the fire, whereas the relative abundance of nitrate-reducing bacteria was significantly lower after burning in the coniferous forests (Fig. S2; Table S9). PERMANOVA also showed significant differences in bacterial functional types between burned and unburned forests for both forest types (R^2^ = 0.640, *P* = 0.001) (Table S10).

Burned and unburned fungal communities were annotated into three trophic modes, which differed in relative abundance between burned and unburned soil samples. The impact of fire on the soil fungal community of the coniferous forest (R^2^ = 0.281, *P* = 0.05) was much greater than that on the fungal community in the broadleaf forest (R^2^ = 0.240, *P* = 0.065) (Table S10). In the coniferous forest, the relative abundances of symbiotrophs and pathotrophs significantly decreased after burning, whereas the relative abundance of saprotrophs significantly increased after the fire (Fig. S3). Based on the trophic mode, fungal communities were further parsed into 78 guilds (Fig. 3B). In the broadleaf forest, the relative abundance of plant pathogens significantly increased after burning, while that of animal pathogens significantly decreased (Table S11). In the coniferous forest, the relative abundances of undefined saprotroph-undefined biotroph and soil saprotroph functional groups significantly increased after burning, while the relative abundances of ectomycorrhizal-fungal parasite, ectomycorrhizal, and animal pathogen functional groups significantly decreased (Table S12). It is noteworthy that the relative abundance of the ectomycorrhizal fungi was significantly higher in the broadleaf forest than in the coniferous forest after burning (Fig. S4; Table S13).

### Soil properties in burned and unburned forests

Our analysis revealed differences in soil physicochemical factors between burned and unburned forests of different types (Fig. S5). Among these factors, SOM, total nitrogen (TN), total phosphorus (TP), and SWC were significantly different between burned and unburned forests and also between the burned coniferous and broadleaf forests (*P* < 0.05). Notably, in the broadleaf forest, these physicochemical factors were significantly reduced after burning, whereas these same physicochemical factors were significantly increased after burning in the coniferous forest. Except for β-glucosidase in the broadleaf forest, there were no significant differences in the activities of the three examined soil extracellular enzymes between burned and unburned forests or between coniferous and broadleaf forests after the fire (*P* < 0.05). Principal component analysis (PCA) based on soil physicochemical factors also revealed a distinct separation between burned and unburned forests and between coniferous and broadleaf forests after burning, indicating larger differences in soil physicochemical factors between forest types after the fire (Fig. S6).

### Correlation between soil microbial communities and soil properties in burned forests

**Fig 4:**
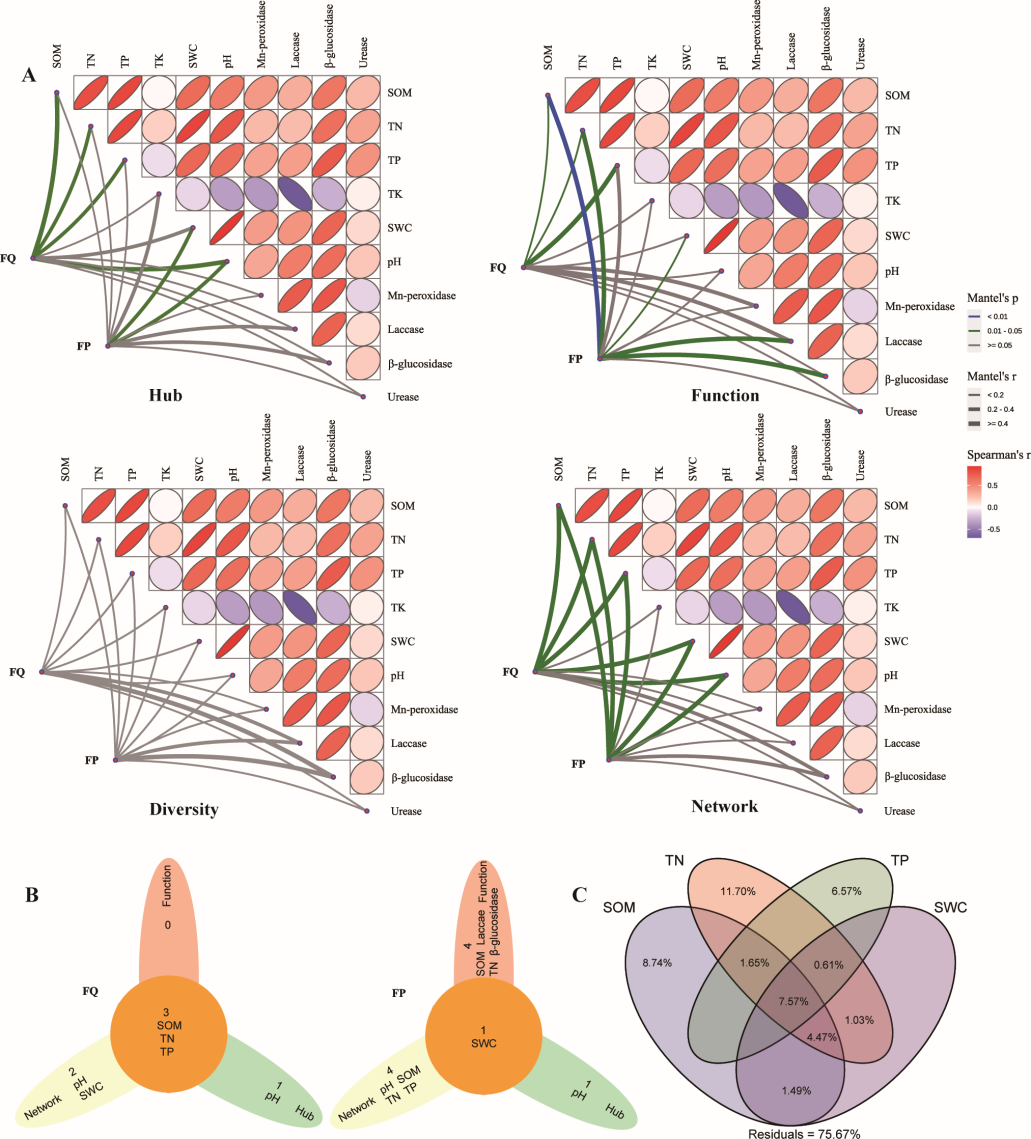
Effect of soil properties on the burned microbial communities. (**A**) Correlation of the burned microbial hub microbiome (genus level), diversity (including ACE richness index, Shannon diversity index, Simpson diversity index, and Chao 1 richness estimator), network, and functional distance matrices with the soil physicochemical factor distance matrices was determined using the Mantel test (based on Spearman's correlation). (**B**) Soil physicochemical factors' similarities and differences that had significant effects on the hub microbiome, network, and function of the burned microbial community, and these defined the same factors as key physicochemical factors impacting the burned microbial community. (**C**) Variance partitioning analysis of key physicochemical factors on the burned microbial hub microbiome. SOM: soil organic matter; TN: total nitrogen; TP: total phosphorus; TK: total potassium; SWC: soil water content.

The correlations of 10 soil physicochemical factors with the burned microbial community were revealed by the Mantel test (Fig. 4A). The results showed that soil physicochemical factors could influence the hub microbiome, function, and network of the burned microbial community but were not significantly correlated with diversity. In the broadleaf forest, SOM, TN, TP, and pH were significantly associated with the hub microbiome; SOM, TN, and TP were associated with function; and SOM, TN, TP, SWC, and pH were associated with the network. In the coniferous forest, SWC and pH were significantly associated with the hub microbiome; SOM, TN, SWC, urease activity, and β-glucosidase activity were associated with function; and SOM, TN, TP, SWC, and pH were associated with the network. Similarities and differences in the physicochemical factors that have important effects on the microbial community in the burned forest were revealed from three levels of the community hub microbiome, function, and network (Fig. 4B). Three, four, and five components of the 10 physicochemical factors were significantly associated with function, the hub microbiome, and the network of the burned microbial community of the broadleaf forest, respectively. Specifically, SOM, TN, and TP, which were all significantly associated with the hub microbiome (R^2^ = 0.173, *P* = 0.013; R^2^ = 0.115, *P* = 0.031; R^2^ = 0.075, *P* = 0.05), function (R^2^ = 0.072, *P* = 0.033; R^2^ = 0.081, *P* = 0.024; R^2^ = 0.166, *P* = 0.022), and network (R^2^ = 0.984, *P* = 0.017; R^2^ = 0.944, *P* = 0.011; R^2^ = 0.626, *P* = 0.017), represent the key physicochemical factors influencing the burned microbial community of the broadleaf forest (Fig. 4). In the coniferous forest, two, five, and five components of the 10 physicochemical factors were significantly associated with the hub microbiome, function, and network of the burned microbial community, respectively. More specifically, TP was significantly associated with the network, urease and β-glucosidase activities were associated with function, pH was significantly associated with both the hub microbiome and the network, and SOM and TN were significantly associated with both function and the network. SWC was significantly associated with the hub microbiome (R^2^ = 0.051, *P* = 0.038), function (R^2^ = 0.133, *P* = 0.031), and network (R^2^ = 0.893, *P* = 0.017), representing the key physicochemical factor influencing the burned microbial community of the coniferous forest (Fig. 4).

We further performed variance partitioning analysis to quantify the contribution of key physicochemical factors to the overall variation in the burned microbial community (Fig. 4C). Four key physicochemical factors were significant (*P*   <  0.001) for interpretation of the burned microbial community and their combined interpretation. Specifically, SOM, TN, TP, and SWC together accounted for 24.33% of the total variation in the burned microbial community, indicating that these factors largely influence the changes in the microbial community of the karst forest after a wildfire.

## DISCUSSION

### Composition and diversity of microbial communities after a wildfire

At the class taxonomic level, the same dominant microbial groups were found in the karst forest before and after burning. Bacteria were mainly composed of Alphaproteobacteria, Gammaproteobacteria, Vicinamibacteria, and Acidobacteriae, and fungi were mainly composed of Agaricomycetes. These groups have also been identified as the common dominant microbial groups in other studies related to burned areas (19, 21). Notably, we found that the relative abundances of Proteobacteria and Acidobacteriota increased after burning in the broadleaf forest, which is consistent with the findings of Yang et al. (22), whereas the relative abundances of these taxa decreased after burning in the coniferous forest, which is also consistent with previous findings (6, 21). The reason for this reduction may be due to the differences in above-ground vegetation types and soil physicochemical properties after a fire in different forest types. In both coniferous and broadleaf forest types, Actinobacteria significantly increased after burning. Actinobacteria can utilize PyOM following a fire to ensure their survival (23). Therefore, our results suggest that bacterial communities undergo different changes according to the forest type in the post-fire karst region.

We found that, after the burning of two types of forests, the relative abundance of Basidiomycota decreased while that of Ascomycota increased, which is consistent with numerous previous studies (4, 22, 24). This phenomenon may be due to the burning off of litter and the stress imposed on mycorrhizal fungi by fire, as well as the chemical inhibition of compounds following a fire (19, 25, 26), which significantly affect the survival of Basidiomycota fungi. Another reason might be that certain classes of Ascomycota fungi such as Leotiomycetes possess thermophilic and heat-resistant properties (4, 27), making them more likely to survive and adapt to post-fire conditions.

This study revealed a slight increase in the α-diversity of microbial communities in the post-fire forest, which is in contrast to previous studies reporting a decrease in α-diversity following forest fires (4, 11). This discrepancy may be attributed to differences in study areas or sampling periods. This study focused on short-term changes in microbial communities in the forest land within the karst rocky desertification area after burning, whereas prior studies primarily conducted long-term monitoring in other regions. Moreover, our β-diversity analysis demonstrated a significant community separation between burned and unburned forest soil microbial communities (*P* < 0.001), indicating that microbial communities in the karst rocky desertification forest region are unlikely to recover to pre-fire levels within 260 days.

### Functions of microbial communities after a wildfire

Our study found that wildfires can cause significant changes in soil microbial community networks. The complexity of the co-occurrence network of microorganisms decreased after the forest fire, which is consistent with the findings of Dai et al. (14) and Yang et al. (19). After a forest fire, the number of microorganisms in the hub microbiome drastically decreased, along with a notable change in the composition of the taxa. This result is attributed to the fact that the hub microbiome in unburned forests is primarily composed of fungi that perform ectomycorrhizal functions. These fungi are a crucial component in maintaining the ecological functions of forest ecosystems, but they are also highly susceptible to the impact of wildfires, including *Scleroderma*, *Rhodanobacter*, and *Nordella* (28–30). Another reason might be that the hub microbiome after a forest fire has specific characteristics such as the ability to survive under extreme environmental conditions and heat resistance. Therefore, the hub microbiome comprises taxa that can not only quickly adapt to the soil environment after a forest fire but can also effectively utilize the nutrients released by the fire to promote their growth and reproduction. Indeed, previous studies confirmed that members of the hub microbiome, including *Occallatibacter*, *Aspergillus*, and *Oidiodendron*, identified after the forest fire in this study have the above characteristics (31–33). In addition, the proportion of all fungi nodes increased after the forest fire, indicating that fungi may play an important role in the short-term succession process in the woodland after a fire. Our results also showed that the complexity of the microbial network and the hub microbiome in the broadleaf forest after a wildfire in the karst region were significantly higher than those in the coniferous forest, indicating that the recovery speed of the broadleaf forest may be faster than that of the coniferous forest in the short-term recovery process after a fire.

The soil microbial functional groups in the forest lands were affected to varying degrees after forest fires. For bacterial communities, the low-abundance functional groups in the two types of forests were more susceptible to the impact of the fire than the high-abundance groups. The low-abundance functional groups underwent a notable shift from being predominantly parasitic before the fire to primarily phototrophic and photoautotrophic after the fire. The high-abundance functional groups were relatively less affected and remained predominantly chemoheterotrophic. This result is consistent with the findings of Cheng et al. (7). This phenomenon may be due to the large-scale destruction of vegetation after the fire, resulting in exposed soil and direct sunlight.

For fungal communities, both high- and low-abundance functional groups were greatly affected after the forest fire possibly because fungal communities are more sensitive to forest fires, as highlighted above. Several studies have also confirmed this effect (15, 18, 19). Additionally, forest fires led to a shift of the fungal symbiotic community toward saprotrophic functional groups. For instance, the relative abundance of ectomycorrhizal fungi decreased after the fire, whereas the relative abundance of saprotrophic fungi significantly increased following the fire. The reason for this difference might be due to the damage or death caused by the burned above-ground vegetation to the underground root systems, thus greatly reducing the symbiotic fungi associated with plant roots. In addition, the dead root systems may recruit or enrich more microorganisms with saprotrophic characteristics. For example, the hub microbiome comprising *Udeniomyces*, *Aspergillus*, and *Chloridium* identified after the forest fire in this study has saprophytic properties, and related studies have confirmed that the hub microbiome not only survives in harsh environments but also has the ability to produce high levels of lignocellulosic enzymes (34–36). Additionally, we found that the functional groups for aromatic compound degradation significantly increased after the forest fire, which may be attributed to the additional input of aromatic compounds into the soil after a fire. Microbial groups capable of utilizing such substances are more likely to adapt to this environment and grow rapidly, such as the hub microbiome comprising *Mycobacterium* and *Aspergillus* identified in this study. Previous research has confirmed the ability of these microorganisms to decompose and transform aromatic compounds (36, 37).

### Effect of karst forest soil physicochemical factors on microbial communities after a wildfire

Wildfires burn a substantial proportion of the vegetation within the woodland and return nutrients to the soil, leading to changes in soil physicochemical factors such as SOM, TN, TP, pH, and β-glucosidase in the forest. We found that SOM, TN, TP, and SWC increased significantly in the coniferous woodland soils after fire, whereas these factors decreased significantly in the burned broadleaf woodland. This may be due to the differences in nutrient mineralization rates between the two forest types following exposure to high-temperature and high-heat conditions, along with the different mechanisms of post-fire woodland restoration (38–40). In addition, there were no significant differences in the activities of the three extracellular enzymes urease, Mn-peroxidase, and laccase between burned and unburned forests. This result differs from previous studies that reported a significant decrease in extracellular enzyme activities after burning (41). This difference may be related to the sampling sites after the fire, as Rasmussen et al. (41) reported significant differences in extracellular enzyme activities at different locations within the same habitat in Mississippi, USA. This difference could also reflect the specific life activities of functional microbial groups in the burned forest, as some members of the identified hub microbiome are efficient producers of these enzymes.

Numerous studies have shown that soil physicochemistry significantly affects the α-diversity of microbial communities after a fire; however, we found no significant correlation between the α-diversity of microbial communities after the fire with the soil physicochemical factors for both forest types. This result may be attributed to the unique climatic environment of the karst region and the greater influence of surface vegetation than soil physicochemical properties during the restoration process (42, 43). Wildfires lead to varying degrees of changes in microbial functions, which can be attributed to changes in the soil physicochemical properties such as the soil moisture, carbon, and nitrogen levels caused by wildfires. This study found that SOM, TN, and SWC significantly affected the function of soil microbial communities. Cheng et al. (44) found that SWC and TN were the main influencing factors of the functional diversity of soil microbes in burn sites of a *Larix gmelinii* forest in a cold temperate zone. Our results thus support these conclusions. Additionally, soil TN and SWC are essential for maintaining various functions and material conversions in soil microorganisms, and significant changes in their contents play a key role in the functional diversity of microbial communities (44–46). We found that the activities of extracellular carbohydrate hydrolases (β-glucosidase and laccase) also significantly influence the function of soil microorganisms in burnt coniferous forests. This phenomenon may be due to microorganisms secreting more hydrolases to break down unstable SOM to help adapt to the post-fire environment and maintain their survival, thereby leading to functional shifts in specific microorganisms.

SOM, TN, TP, and SWC were simultaneously and significantly associated with the hub microbiome, functions, and networks and were defined as the key soil physiochemical factors impacting post-fire microbial communities in karst regional woodlands. SOM is an important carbon reservoir and nutrient for post-fire microbial metabolism, which plays an important role in soil carbon and nutrient cycling (47, 48). VanderRoest et al. (16) simulated severe wildfires through a controlled “pyrocosm” approach and identified that post-fire biodegradable SOM was enriched with heterotrophic microbes and also contained many substrates that support microbial metabolism. In addition, the decomposition and mineralization of SOM by post-fire microorganisms are also inseparable from TN and TP (14, 17), whereas SWC is an important predictor of the post-fire soil microbial community composition (11, 13, 33). Our study also found a significant positive correlation among these four key soil physicochemical factors (Fig. S6B). This indicates that the recovery of post-fire microorganisms in the karst forests is a result of the synergistic effects of multiple soil physicochemical factors.

### Conclusions

This study revealed the composition, diversity, function, and network of microbial communities after a wildfire in different forest types of the karst ecosystem, demonstrating that soil physicochemical factors can affect the microbial communities after fire to varying degrees. SOM, TN, TP, and SWC have significant impacts on the composition, function, and network of post-fire microbial communities and were identified as the key soil physicochemical factors influencing the short-term recovery of microbial communities in the woodland after a fire in the karst region. These findings can provide certain guidance for the restoration and management of post-fire soil ecosystems in the karst forest region. However, this study only provides characteristics of the microbial communities in the short-term post-fire stage in the karst forest region. Future studies should focus on long-term site-based monitoring to gain a deeper understanding of the succession patterns and recovery mechanisms of the post-fire microbial community.

## MATERIALS AND METHODS

### Study sites and experimental design

The study sites are located in Huishui County, Southern Guizhou Province, China, which is part of a typical karstic rocky, severe desertification area in the province. The region lies within the area of influence of subtropical monsoons, with an average annual temperature ranging from 14°C to 16°C. The mean annual precipitation is 1,213.4 mm, and both water and heat are present simultaneously during the same season. The soils at the selected sampling locations are classified as loess forest soils. From February to April 2023, forest fires were frequent in the area, with a total burned area of 231.42 ha. The main burned tree species were *Quercus fabri* and *Pinus massoniana*; therefore, we selected pure *Q. fabri* and *P. massoniana* forests as our focus for this study.

Soil samples were collected on 1 November 2023 from four distinct forest types: unburned *Q. fabri* forest (CQ), burned *Q. fabri* forest (FQ) (the fire occurred on 13 February 2023), unburned *P. massoniana* forest (CP), and burned *P. massoniana* forest (FP) (the fire occurred on 11 February 2023). According to the classification of fire severity levels by Brown et al. (25), the burned sample sites in this study belong to a high severity level (litter fully consumed, duff almost completely consumed, shrub layer completely consumed or charred main stems remaining, overstory trees charred to full tree height and canopy needles/leaves mostly to completely consumed). Unburned reference plots were positioned as close as possible to the burned plots. A total of 20 plots were selected, each with an area of approximately 100 m^2^, including five plots for each forest type. The sampling and location details are provided in Table S1.

### Soil sampling

At each plot, we established a 10 × 10 m square plot with 3 × 3 m subplots at the four corners and the center. In each subplot, sampling was performed randomly along an S-shape. All researchers wore sterile gloves to carefully remove any litter and the organic layer, focusing on removing any ash, charcoal, and debris on the surface as much as possible. Five soil cores from the top 10 cm of the mineral layer were collected with an auger (inner diameter of 5 cm; sterilized with 10% bleach followed by 75% ethanol). All soil cores from the five subplots were pooled together into one composite sample (i.e., one replicate). Finally, all composite soil samples were placed in plastic zipper bags, chilled on ice, and transported to the laboratory within 12 h.

Prior to analysis, all samples were meticulously sieved through a 2-mm sieve after the removal of visible stones, roots, and organic remnants. Processed samples were divided into three sub-samples. The first sub-sample was immediately frozen in liquid nitrogen and stored at −80°C until used for microbial DNA extraction. The second sub-sample was stored at 4°C for subsequent measurement of Mn-peroxidase, laccase, β-glucopyranoside, and urease enzyme activities. The last sub-sample was air-dried and maintained at room temperature to measure the SWC, pH, SOM, total nitrogen (TN), total phosphorus (TP), and total potassium (TK) levels.

### Measurements of soil properties

The activities of urease, β-glucosidase, Mn-peroxidase, and laccase were determined using assay kits obtained from Beijing Solarbio & Technology Co., Ltd. (https://www.solarbio.com/) based on the optical density values measured using a Multiskan SkyHigh microplate absorbance reader. SWC was measured gravimetrically as described previously (49). Soil pH was measured in a 1:2.5 soil/water suspension using a FiveEasy Plus pH meter (50). SOM was analyzed by the dichromate oxidation method with the external heating procedure. TN was determined by the Kjeldahl method (51), TP was determined by the sulfuric acid digestion procedure, and TK was detected by NaOH melting and determined by flame photometry (52). Three replicate measurements were taken for each sample.

### DNA extraction and amplicon sequencing

DNA was extracted from 0.5 g homogeneous soil samples using the TGuide S96 Magnetic Kit for Soil (Tiangen Biotech, Beijing, China) according to the manufacturer's instructions. Each soil sample was extracted three times, and equal amounts of DNA were taken to create a composite DNA sample (10 µL) for amplification. The bacterial 16S rRNA gene V3–V4 region was amplified using the primers 338F (5′-ACTCCTACGGGAGGCAGCA-3′) and 806R (5′-GGACTACHVGGGTWTCTAAT-3′), and the fungal ITS1 region was amplified using the primers ITS1 (5′-CTTGGTCATTTAGAGGAAGTAA-3′) and ITS2 (5′-GCTGCGTTCTTCATCGATGC-3′). Polymerase chain reaction (PCR) was performed in a total reaction volume of 10 µL. The specific amplification system and temperature cycling parameters were consistent with those described by Yang et al. (19). Each sample was amplified three times and then mixed. Subsequently, the total PCR amplicons were purified using Agencourt AMPure XP Beads (Beckman Coulter, Indianapolis, IN, USA) and the constructed library was assessed using a Qubit dsDNA HS Assay Kit and Qubit 4.0 fluorometer (Invitrogen, Thermo Fisher Scientific, Eugene, OR, USA). After the individual quantification step, amplicons were pooled in equal amounts. Finally, the resulting PCR products were subjected to high-throughput amplicon sequencing using an Illumina novaseq 6000 platform (Illumina, San Diego CA, USA) at Biomarker Technologies Co. (Beijing, China).

### Bioinformatic analysis

Raw reads obtained from sequencing were quality-filtered by Trimmomatic 0.33 (53). Subsequently, the software Cutadapt 1.9.1 was used to identify and remove primer sequences according to parameters allowing a maximum mismatch rate of 20% and a minimum coverage of 80%. The obtained clean reads were then de-replicated and de-noised using the DADA2 default parameter in QIIME2 (54). Finally, paired-end sequences were merged using USEARCH 10 (the reads of each sample were merged according to a minimum overlap length of 10 bp, a minimum similarity of 90% allowed in the overlap region, and a maximum number of mismatched bases of 5 bp) and chimeras were removed using UCHIME 8.1 (55). Through the above process, amplicon sequence variants (ASVs) were generated, with a conservative threshold for ASV filtering set at 0.005% (56).

Taxonomic identification was performed according to the SILVA (release 138) and UNITE (release 8.0) reference databases for the classification and annotation of bacterial and fungal ASVs, respectively, with a confidence threshold of 70%. In addition, bacterial functional profiles were predicted using functional annotation of prokaryotic taxa (FAPROTAX) (57) and fungal functional guilds were inferred using the program FUNGuild (58).

### Data analyses

Burned and unburned microbial community data were subjected to the non-parametric Wilcoxon rank-sum test based on the ggpubr package in R 4.3.2. Principal component analysis (PCA) and permutational multivariate analysis of variance (PERMANOVA) were performed based on the vegan R package. Co-occurrence network analysis between burned and unburned soil microbiota was performed based on Spearman's correlation coefficients (r_s_ > 0.8 or <–0.8; *P* < 0.01) using the psych and WGCNA packages in R. Fungal and bacterial ASVs present in 70% of the samples were retained for the network analysis, and visualization and calculation of topographical network parameters were conducted in Gephi. Subsequently, the hub microbiome was determined according to the network topological characteristics of degree and closeness centrally (56, 59). Burned and unburned soil properties were also subjected to the non-parametric Wilcoxon rank-sum test, principal coordinate analysis (PCoA), and correlation analysis based on the vegan, ggpubr, and ggcorb R packages. In addition, correlation analyses of burned and unburned soil properties and microbial community data, including the Mantel test and variance partitioning analysis, were performed based on the vegan package in R.

## Data Availability

The sequence data generated in this study have been uploaded to the National Center for Biotechnology Information Sequence Read Archive database (BioProject PRJNA1122071).

## SUPPLEMENTAL MATERIAL

Supplemental material aem.01245-24-s0001.docx Tables S1 to S13; Figures S1 to S6.

ASM does not own the copyrights to Supplemental Material that may be linked to, or accessed through, an article. The authors have granted ASM a non-exclusive, world-wide license to publish the Supplemental Material files. Please contact the corresponding author directly for reuse.
