# Supplementary material for: Effects of wildfire on soil microbial communities in karst forest ecosystems of southern Guizhou Province, China
Source: Appl Environ Microbiol. 2024 Oct 30;90(11):e01245-24. doi: 10.1128/aem.01245-24 (PMC11577766; doi:10.1128/aem.01245-24)
Supplement: Supplemental material — Tables S1 to S13; Figures S1 to S6. [file aem.01245-24-s0001.docx]

**Supplementary materials**

**Table S1.** General description of the control and burned sites. CQ: Unburned *Quercus fabri* forest; FQ: Burned *Quercus fabri* forest; CP: Unburned *Pinus massoniana* forest; FP: Burned *Pinus massoniana* forest.

| Site | CQ | FQ | CP | FP |
| --- | --- | --- | --- | --- |
| Burned year | Unburned | 2023 | Unburned | 2023 |
| Type | *Quercus fabri* | *Quercus fabri* | *Pinus massoniana* | *Pinus massoniana* |
| Vegetation type | Arbor (*Quercus fabri*) | Herbage (*Imperata cylindrica*) | Arbor (*Pinus massoniana*) | Herbage (*Pteridium aquilinum*) |
| Latitude(N) | 26°2′44′′ | 26°2′51′′ | 26°4′57′′ | 22°4′54′′ |
| Longitude(E) | 106°53′13′′ | 106°53′10′′ | 106°39′48′′ | 106°39′56′′ |
| Aspect | Sunny | Sunny | Sunny | Sunny |

**Table S2.** Raw reads, high-quality reads and ASV numbers obtained from amplification sequencing of 20 soil samples.

| sample ID | bacteria | | | fungi | | |
| --- | --- | --- | --- | --- | --- | --- |
|  | raw reads | high-quality reads | ASV numbers | raw reads | high-quality reads | ASV numbers |
| FQ1 | 80205 | 72504 | 2702 | 80070 | 66738 | 776 |
| FQ2 | 79991 | 72216 | 2275 | 80124 | 68836 | 708 |
| FQ3 | 79919 | 72168 | 2739 | 80078 | 69800 | 862 |
| FQ4 | 80092 | 72112 | 2459 | 79850 | 67642 | 852 |
| FQ5 | 79987 | 73023 | 2834 | 80071 | 67850 | 690 |
| FP1 | 79812 | 72828 | 2792 | 79901 | 68029 | 804 |
| FP2 | 80003 | 72300 | 2063 | 80124 | 69123 | 509 |
| FP3 | 80115 | 72539 | 3123 | 79852 | 62092 | 530 |
| FP4 | 80142 | 73995 | 2407 | 80244 | 68236 | 816 |
| FP5 | 79873 | 72190 | 2241 | 80004 | 68304 | 663 |
| CQ1 | 79948 | 72662 | 2986 | 80126 | 69813 | 746 |
| CQ2 | 79934 | 73164 | 3004 | 79986 | 70004 | 795 |
| CQ3 | 80010 | 72060 | 2853 | 79951 | 70069 | 605 |
| CQ4 | 80051 | 72925 | 2945 | 79851 | 70069 | 764 |
| CQ5 | 80083 | 73120 | 2704 | 79968 | 71159 | 751 |
| CP1 | 43712 | 39567 | 1705 | 79964 | 70391 | 575 |
| CP2 | 80013 | 72454 | 2630 | 79990 | 70747 | 562 |
| CP3 | 79864 | 72317 | 2218 | 79918 | 69575 | 660 |
| CP4 | 80010 | 72245 | 2144 | 79893 | 71051 | 478 |
| CP5 | 80235 | 72799 | 2553 | 79886 | 52857 | 649 |

**Table S3.** The relative abundance of Class in soil bacterial and fungal communities between burned and unburned. Low abundance classes with a total sequence number of less than 1% in all samples were classified as “Other”. Values in parentheses represents the standard error of the mean with five replicates. Different lowercase letters indicate significant differences among sites (p< 0.05). Abbreviations used are as follows: Unburned *Quercus fabri* forest (CQ), Burned *Quercus fabri* forest (FQ), Unburned *Pinus massoniana* forest (CP), Burned *Pinus massoniana* forest (FP).

|  | FQ | CQ | FP | CP | *p-values* |
| --- | --- | --- | --- | --- | --- |
| Bacteria |  |  |  |  |  |
| Alphaproteobacteria | 20.18(1.61)b | 15.92(1.09)a | 19.73(1.60)ab | 22.62(5.26)b | **0.018** |
| Gammaproteobacteria | 9.75(1.61)a | 12.58(1.03)b | 12.28(1.67)b | 14.46(2.10)b | **0.003** |
| Acidobacteriota | 14.70(4.99)b | 7.69(1.04)a | 6.48(5.04)a | 2.63(0.66)a | **0.001** |
| Vicinamibacteria | 3.65(2.13)a | 7.30(0.56)b | 7.07(2.45)b | 13.56(3.25)c | **0.000** |
| Actinobacteria | 7.31(1.59)b | 3.70(0.77)a | 10.96(3.08)c | 5.07(2.54)ab | **0.000** |
| Bacteroidia | 5.77(1.25)a | 6.51(1.25)ab | 8.55(2.51)b | 5.72(0.85)a | **0.041** |
| unclassified_Bacteria | 2.78(0.46)a | 5.96(0.88)b | 3.09(1.08)a | 5.86(1.17)b | **0.000** |
| Verrucomicrobiae | 3.78(0.76)c | 6.94(0.38)d | 1.84(0.29)a | 2.42(0.88)a | **0.000** |
| Thermoleophilia | 4.32(0.47)a | 3.76(0.74)a | 3.13(1.36)a | 3.07(0.95)a | 0.162 |
| Blastocatellia | 3.38(1.25)a | 2.99(0.43)a | 3.30(1.17)a | 3.92(1.56)a | 0.663 |
| Acidimicrobiia | 2.16(0.24)a | 2.83(0.28)ab | 2.84(0.52)ab | 3.17(1.07)b | 0.117 |
| Polyangia | 2.12(0.36)a | 2.73(0.28)a | 2.79(0.48)a | 2.19(0.87)a | 0.143 |
| Planctomycetes | 3.65(1.28)b | 2.60(0.30)ab | 1.78(0.85)a | 1.65(0.74)a | **0.008** |
| Gemmatimonadetes | 2.30(0.63)b | 1.27(0.16)a | 2.20(0.86)b | 1.43(0.24)a | **0.019** |
| Anaerolineae | 1.54(0.76)a | 2.71(0.74)b | 1.46(0.85)a | 1.39(0.91)a | 0.065 |
| Methylomirabilia | 0.79(0.64)a | 4.05(0.61)b | 0.51(0.86)a | 1.36(0.75)a | **0.000** |
| Saccharimonadia | 2.22(0.48)b | 0.59(0.17)a | 2.13(1.12)b | 0.34(0.17)a | **0.000** |
| unclassified_Chloroflexi | 0.81(0.49)ab | 1.68(0.42)c | 0.65(0.32)a | 1.30(0.31)b | **0.003** |
| Myxococcia | 0.84(0.24)a | 1.42(0.12)b | 0.49(0.25)a | 1.55(0.72)b | **0.002** |
| others | 7.95(0.79)ab | 6.75(0.64)a | 8.73(2.21)b | 6.28(1.19)a | **0.043** |
| Fungi |  |  |  |  |  |
| Agaricomycetes | 46.44(16.59)a | 71.01(11.72)b | 41.40(9.78)a | 66.95(13.87)b | **0.005** |
| Sordariomycetes | 11.23(3.56)a | 7.89(4.42)a | 11.76(3.33)a | 9.07(3.42)a | 0.340 |
| unclassified_Fungi | 12.92(12.12)b | 5.18(6.90)ab | 4.62(2.22)ab | 0.96(0.52)a | 0.093 |
| Pezizomycetes | 1.91(1.95)a | 1.33(1.69)a | 4.82(8.32)a | 10.62(12.39)a | 0.234 |
| Dothideomycetes | 6.73(1.99)bc | 2.93(1.88)ab | 8.54(5.29)c | 1.59(0.59)a | **0.007** |
| Eurotiomycetes | 3.79(1.43)b | 1.79(0.64)a | 4.46(1.54)b | 3.91(1.36)b | **0.025** |
| unclassified_Ascomycota | 3.71(2.35)ab | 1.67(1.05)a | 5.78(4.19)b | 0.78(0.22)a | **0.024** |
| Leotiomycetes | 3.10(1.80)a | 3.55(3.03)a | 2.30(1.57)a | 2.14(1.50)a | 0.676 |
| Mortierellomycetes | 2.08(1.03)a | 2.21(1.23)a | 1.02(0.83)a | 1.43(0.76)a | 0.222 |
| unidentified | 1.40(0.58)a | 0.70(0.39)a | 3.35(2.21)b | 0.82(0.38)a | **0.009** |
| Archaeorhizomycetes | 1.63(3.02)a | 0.10(0.08)a | 4.96(6.45)a | 0.02(0.02)a | 0.139 |
| Others | 5.06(1.98)b | 1.65(0.62)a | 7.00(3.08)b | 1.71(0.44)a | **0.001** |

**Table S4.** Significance analysis of fire on soil microbial community composition. Significant *p* <0.05 are bolded. CQ: Unburned *Quercus fabri* forest; FQ: Burned *Quercus fabri* forest; CP: Unburned *Pinus massoniana* forest; FP: Burned *Pinus massoniana* forest.

| Group | Bacteria | | Fungi | |
| --- | --- | --- | --- | --- |
|  | R*^2^* | *p* | R*^2^* | *p* |
| All site | 0.341 | **0.001** | 0.333 | **0.001** |
| FQ/CQ | 0.273 | **0.001** | 0.288 | **0.001** |
| FP/CP | 0.025 | **0.001** | 0.192 | **0.001** |
| FQ/FP | 0.209 | **0.001** | 0.271 | **0.001** |
| CQ/CP | 0.243 | **0.001** | 0.214 | **0.007** |

**Table S5.** The hub microbiome in soil microbial community after fire disturbance. CQ: Unburned *Quercus fabri* forest; FQ: Burned *Quercus fabri* forest.

| FQ | | CQ | |
| --- | --- | --- | --- |
| OUTID | Genus | OUTID | Genus |
| ASV16 | *Roseiarcus* | ASV110 | *Bryobacter* |
| ASV66 | *unclassified_Acidimicrobiia* | ASV137 | *Flavobacterium* |
| ASV73 | *unclassified_Acidobacteriaceae*  *__Subgroup_1* | ASV145 | *Flavobacterium* |
| ASV93 | *Mycobacterium* | ASV161 | *unclassified_Xanthobacteraceae* |
| ASV112 | *unclassified_Acidobacteriales* | ASV170 | *Gemmatimonas* |
| ASV139 | *unclassified_Micropepsaceae* | ASV182 | *uncultured_Desulfovirga_sp.* |
| ASV158 | *unclassified_Caulobacteraceae* | ASV322 | *Allorhizobium_Neorhizobium_*  *Pararhizobium_Rhizobium* |
| ASV166 | *Occallatibacter* | ASV340 | *unclassified_Comamonadaceae* |
| ASV343 | *Methylobacterium_*  *Methylorubrum* | ASV388 | *Gaiella* |
| ASV359 | *Bryobacter* | ASV433 | *Reyranella* |
| ASV366 | *unclassified_Blastocatellaceae* | ASV454 | *unclassified_Chitinophagaceae* |
| ASV485 | *unclassified_Ilumatobacteraceae* | ASV488 | *unclassified_Acidobacteriales* |
| ASV633 | *uncultured_proteobacterium* | ASV534 | *unclassified_Rhizobiaceae* |
| ASV1373 | *unclassified_Cyanobacteriales* | ASV582 | *uncultured_gamma_proteobacterium* |
| ASV3844 | *unclassified_TRA3_20* | ASV769 | *Terrimonas* |
| ASV214 | *unclassified_Basidiomycota* | ASV944 | *unclassified_Anaerolineaceae* |
| ASV247 | *Chloridium* | ASV1068 | *unclassified_Microtrichales* |
| ASV266 | *Phialocephala* | ASV1193 | *unclassified_AKYH767* |
| ASV328 | *unclassified_Ascomycota* | ASV3910 | *unclassified_Chitinophagaceae* |
| ASV365 | *Udeniomyces* | ASV5521 | *Candidatus_Udaeobacter* |
| ASV710 | *unclassified_Clavicipitaceae* | ASV5552 | *Candidatus_Solibacter* |
| ASV713 | *unclassified_Helotiales* | ASV5586 | *Parafilimonas* |
| ASV855 | *Oidiodendron* | ASV5600 | *unclassified_Vicinamibacterales* |
| ASV964 | *Articulospora* | ASV5601 | *unclassified_Acidobacteriales* |
|  |  | ASV5644 | *Haliangium* |
|  |  | ASV5697 | *unclassified_Acidobacteriales* |
|  |  | ASV5698 | *unclassified_Chitinophagaceae* |
|  |  | ASV5706 | *Candidatus_Udaeobacter* |
|  |  | ASV5717 | *unclassified_Chitinophagaceae* |
|  |  | ASV5797 | *Ellin6067* |
|  |  | ASV5814 | *unclassified_Chloroflexi* |
|  |  | ASV5832 | *Terrimonas* |
|  |  | ASV5876 | *Nordella* |
|  |  | ASV5941 | *Variovorax* |
|  |  | ASV5947 | *unclassified_Anaerolineae* |
|  |  | ASV5966 | *unclassified_Pedosphaeraceae* |
|  |  | ASV10925 | *P3OB_42* |
|  |  | ASV10944 | *uncultured_Acidobacteria_bacterium* |
|  |  | ASV12306 | *unclassified_Bacteria* |
|  |  | ASV13586 | *unclassified_Rokubacteriales* |
|  |  | ASV13800 | *unclassified_Elsterales* |
|  |  | ASV13881 | *Pedomicrobium* |
|  |  | ASV14518 | *unclassified_Bacteria* |
|  |  | ASV26498 | *unclassified_Vicinamibacterales* |
|  |  | ASV26496 | *unclassified_KF_JG30_B3* |
|  |  | ASV26515 | *uncultured_Candidatus_*  *Rokubactera_bacterium* |
|  |  | ASV26525 | *unclassified_A4b* |
|  |  | ASV26560 | *unclassified_JG30_KF_AS9* |
|  |  | ASV26565 | *unclassified_Vicinamibacteraceae* |
|  |  | ASV26591 | *MND1* |
|  |  | ASV26602 | *unclassified_SC_I_84* |
|  |  | ASV26611 | *unclassified_Vicinamibacterales* |
|  |  | ASV26630 | *Steroidobacter* |
|  |  | ASV26726 | *unclassified_Comamonadaceae* |
|  |  | ASV26754 | *uncultured_Hyphomicrobiaceae*  *_bacterium* |
|  |  | ASV26780 | *Ellin6067* |
|  |  | ASV26795 | *unclassified_Subgroup_17* |
|  |  | ASV26829 | *uncultured_Acidobacteriales_bacterium* |
|  |  | ASV26864 | *unclassified_Desulfobacterota* |
|  |  | ASV26918 | *Jatrophihabitans* |
|  |  | ASV26936 | *unclassified_Chitinophagaceae* |
|  |  | ASV26989 | *unclassified_Bacteria* |
|  |  | ASV27070 | *Flavisolibacter* |
|  |  | ASV27078 | *Candidatus_Udaeobacter* |
|  |  | ASV27098 | *unclassified_SC_I_84* |
|  |  | ASV27136 | *unclassified_Vicinamibacteraceae* |
|  |  | ASV27163 | *unclassified_Bacteria* |
|  |  | ASV27177 | *Mycobacterium* |
|  |  | ASV27237 | *Ahniella* |
|  |  | ASV29773 | *unclassified_A21b* |
|  |  | ASV29777 | *Candidatus_Xiphinematobacter* |
|  |  | ASV29816 | *Candidatus_Udaeobacter* |
|  |  | ASV29820 | *unclassified_SC_I_84* |
|  |  | ASV29974 | *unclassified_Saprospiraceae* |
|  |  | ASV29979 | *Rhodanobacter* |
|  |  | ASV30167 | *unclassified_JG30_KF_CM45* |
|  |  | ASV30575 | *Sumerlaea* |
|  |  | ASV18 | *Aspergillus* |
|  |  | ASV767 | *unidentified* |
|  |  | ASV1038 | *Exophiala* |
|  |  | ASV1071 | *Talaromyces* |
|  |  | ASV1518 | *unidentified* |
|  |  | ASV2457 | *Tomentella* |
|  |  | ASV3222 | *Metapochonia* |
|  |  | ASV3867 | *Lecanicillium* |
|  |  | ASV6412 | *Scleroderma* |
|  |  | ASV6414 | *unclassified_Xylariales* |
|  |  | ASV6617 | *unclassified_unidentified* |
|  |  | ASV7064 | *unclassified_Trechisporales* |
|  |  | ASV7066 | *Tuber* |
|  |  | ASV7165 | *Mollisina* |

**Table S6.** The hub microbiome in soil microbial community after fire disturbance. CP: Unburned *Pinus massoniana* forest; FP: Burned *Pinus massoniana* forest.

| FP |  | CP |  |
| --- | --- | --- | --- |
| OUT ID | Genus | OUT ID | Genus |
| ASV5513 | *Aridibacter* | ASV5665 | *Reyranella* |
| ASV10942 | *Flavisolibacter* | ASV5850 | *unclassified_SC_I_84* |
| ASV891 | *Solicoccozyma* | ASV8653 | *P3OB_42* |
| ASV3831 | *Gyrothrix* | ASV13517 | *unclassified_Blastocatellaceae* |
| ASV3902 | *Aspergillus* | ASV13550 | *unclassified_A4b* |
|  |  | ASV13607 | *unclassified_SC_I_84* |
|  |  | ASV13659 | *unclassified_Methyloligellaceae* |
|  |  | ASV13755 | *uncultured_bacterium_gp6* |
|  |  | ASV13935 | *unclassified_JG30_KF_CM45* |
|  |  | ASV13968 | *unclassified_Vicinamibacteraceae* |
|  |  | ASV13991 | *uncultured_gamma_proteobacterium* |
|  |  | ASV14446 | *unclassified_Anaerolineae* |
|  |  | ASV16606 | *RB41* |
|  |  | ASV27407 | *Parafilimonas* |
|  |  | ASV30142 | *Ensifer* |
|  |  | ASV41023 | *unclassified_Vicinamibacteraceae* |
|  |  | ASV41066 | *P3OB_42* |
|  |  | ASV41073 | *uncultured_Candidatus_Rokubactera*  *_bacterium* |
|  |  | ASV41097 | *unclassified_Rokubacteriales* |
|  |  | ASV41180 | *BD1_7_clade* |
|  |  | ASV41302 | *unclassified_Alphaproteobacteria* |
|  |  | ASV42653 | *unclassified_Vicinamibacteraceae* |
|  |  | ASV42660 | *Subgroup_10* |
|  |  | ASV42683 | *RB41* |
|  |  | ASV42689 | *unclassified_Vicinamibacterales* |
|  |  | ASV42768 | *unclassified_Bacteria* |
|  |  | ASV42807 | *unclassified_Bacteria* |
|  |  | ASV42878 | *Ahniella* |
|  |  | ASV42888 | *unclassified_Alphaproteobacteria* |
|  |  | ASV42972 | *unclassified_Chloroflexi* |
|  |  | ASV43144 | *Ahniella* |
|  |  | ASV2543 | *Staphylotrichum* |
|  |  | ASV6347 | *Chaetopsina* |
|  |  | ASV7897 | *unclassified_Helotiales* |
|  |  | ASV9610 | *Penicillium* |
|  |  | ASV9614 | *Entoloma* |

**Table S7.** Differences in bacterial community function (top 10 in abundance) between FQ and CQ were determined using nonparametric wilcoxon rank sum test (10 pairs of functional characteristics). CQ: Unburned *Quercus fabri* forest; FQ: Burned *Quercus fabri* forest

| Comparison | Taxa | FQ  (mean ± sd) | CQ  (mean ± sd) | *P-values* | Significance |
| --- | --- | --- | --- | --- | --- |
| FQ - CQ | Chemoheterotrophy | 32.53 (2.06) | 30.11 (1.38) | 0.0556 | ns |
| FQ - CQ | Aerobic_chemoheterotrophy | 31.54 (2.07) | 29.00 (1.55) | 0.0556 | ns |
| FQ - CQ | Nitrogen_fixation | 4.04 (0.92) | 4.92 (0.66) | 0.0952 | ns |
| FQ - CQ | Predatory_or_exoparasitic | 2.46 (0.90) | 4.29 (0.69) | **0.0317** | * |
| FQ - CQ | Phototrophy | 3.55 (0.75) | 1.61 (0.29) | **0.0079** | ** |
| FQ - CQ | Photoautotrophy | 2.99 (0.99) | 1.55 (0.31) | **0.0317** | * |
| FQ - CQ | Ureolysis | 1.59 (0.60) | 1.41 (0.45) | 0.6905 | ns |
| FQ - CQ | Nitrate_reduction | 0.89 (0.33) | 1.64 (0.22) | **0.0079** | ** |
| FQ - CQ | Aromatic_compound_degradation | 1.64 (0.50) | 1.24 (0.52) | 0.2222 | ns |
| FQ - CQ | Animal_parasites_or_symbionts | 1.45 (0.10) | 3.66 (0.78) | **0.0079** | ** |

**Table S8.** Differences in bacterial community function (top 10 in abundance) between FP and CP were determined using nonparametric wilcoxon rank sum test (10 pairs of functional characteristics). CP: Unburned *Pinus massoniana* forest; FP: Burned *Pinus massoniana* forest.

| Comparison | Taxa | FP  (mean ± sd) | CP  (mean ± sd) | *P-values* | Significance |
| --- | --- | --- | --- | --- | --- |
| FP - CP | Chemoheterotrophy | 31.77 (3.47) | 29.34 (2.95) | 0.2222 | ns |
| FP - CP | Aerobic_chemoheterotrophy | 29.87 (4.12) | 26.36 (3.68) | 0.2222 | ns |
| FP - CP | Nitrogen_fixation | 1.68 (0.82) | 3.43 (1.29) | **0.0317** | * |
| FP - CP | Predatory_or_exoparasitic | 2.44 (0.97) | 1.95 (0.99) | 0.6905 | ns |
| FP - CP | Phototrophy | 1.90 (0.42) | 2.41 (0.59) | 0.3095 | ns |
| FP - CP | Photoautotrophy | 1.81 (0.42) | 2.34 (0.56) | 0.1508 | ns |
| FP - CP | Ureolysis | 2.96 (1.78) | 1.28 (0.52) | 0.1508 | ns |
| FP - CP | Nitrate_reduction | 2.11 (0.57) | 2.93 (0.56) | 0.1508 | ns |
| FP - CP | Aromatic_compound_degradation | 3.03 (1.07) | 0.73 (0.33) | **0.0079** | ** |
| FP - CP | Animal_parasites_or_symbionts | 1.44 (0.75) | 1.07 (0.38) | 0.3095 | ns |

**Table S9.** Differences in bacterial community function (top 10 in abundance) between FQ and FP were determined using nonparametric wilcoxon rank sum test (10 pairs of functional characteristics). FQ: Burned *Quercus fabri* forest; FP: Burned *Pinus massoniana* forest.

| Comparison | Taxa | FQ  (mean ± sd) | FP  (mean ± sd) | *P-values* | Significance |
| --- | --- | --- | --- | --- | --- |
| FQ - FP | Chemoheterotrophy | 32.53 (2.06) | 31.77 (3.47) | 0.6905 | ns |
| FQ - FP | Aerobic_chemoheterotrophy | 31.54 (2.07) | 29.87 (4.12) | 0.5476 | ns |
| FQ - FP | Nitrogen_fixation | 4.04 (0.92) | 1.68 (0.82) | **0.0159** | * |
| FQ - FP | Predatory_or_exoparasitic | 2.46 (0.90) | 2.44 (0.97) | 1.0000 | ns |
| FQ - FP | Phototrophy | 3.55 (0.75) | 1.90 (0.42) | **0.0079** | ** |
| FQ - FP | Photoautotrophy | 2.99 (0.99) | 1.81 (0.42) | **0.0317** | * |
| FQ - FP | Ureolysis | 1.59 (0.60) | 2.96 (1.78) | 0.2222 | ns |
| FQ - FP | Nitrate_reduction | 0.89 (0.33) | 2.11 (0.57) | **0.0079** | ** |
| FQ - FP | Aromatic_compound_degradation | 1.64 (0.50) | 3.03 (1.07) | 0.0556 | ns |
| FQ - FP | Animal_parasites_or_symbionts | 1.45 (0.10) | 1.44 (0.75) | 0.2222 | ns |

**Table S10.** Significance analysis of fire on soil microbial community function. Significant *p* <0.05 are bolded. CQ: Unburned *Quercus fabri* forest; FQ: Burned *Quercus fabri* forest; CP: Unburned *Pinus massoniana* forest; FP: Burned *Pinus massoniana* forest.

| Group | Bacteria | | Fungi | |
| --- | --- | --- | --- | --- |
|  | R*^2^* | *p* | R*^2^* | *p* |
| All site | 0.640 | **0.001** | 0.296 | **0.016** |
| FQ/CQ | 0.722 | **0.009** | 0.240 | 0.065 |
| FP/CP | 0.304 | **0.043** | 0.281 | **0.05** |
| FQ/FP | 0.263 | 0.075 | 0.247 | **0.035** |
| CQ/CP | 0.652 | **0.009** | 0.128 | 0.348 |

**Table S11.** Differences in fungal community function (top 10 in abundance) between FQ and CQ were determined using nonparametric wilcoxon rank sum test (10 pairs of functional characteristics). CQ: Unburned *Quercus fabri* forest; FQ: Burned *Quercus fabri* forest

| Comparison | Taxa | FQ  (mean ± sd) | CQ  (mean ± sd) | *P-values* | Significance |
| --- | --- | --- | --- | --- | --- |
| FQ - CQ | Ectomycorrhizal | 46.09 (21.83) | 65.92 (21.05) | 0.2222 | ns |
| FQ - CQ | Undefined_Saprotroph | 13.06 (5.48) | 13.70 (11.45) | 1.0000 | ns |
| FQ - CQ | Ectomycorrhizal-Fungal_Parasite | 0.18 (0.19) | 0.08 (0.10) | 0.3457 | ns |
| FQ - CQ | Ectomycorrhizal-Undefined_Saprotroph | 15.09 (19.32) | 2.98 (2.53) | 0.8413 | ns |
| FQ - CQ | Undefined_Saprotroph-Undefined_Biotroph | 0.08 (0.08) | 0.02 (0.03) | 0.1612 | ns |
| FQ - CQ | Soil_Saprotroph | 4.20 (8.07) | 3.39 (7.05) | 0.8413 | ns |
| FQ - CQ | Plant_Pathogen | 3.25 (1.74) | 1.48 (1.09) | 0.0317 | * |
| FQ - CQ | Wood_Saprotroph | 0.84 (1.35) | 1.44 (1.09) | 0.3095 | ns |
| FQ - CQ | Animal_Pathogen | 0.49 (0.23) | 3.70 (3.50) | 0.0159 | * |
| FQ - CQ | Animal_Pathogen-Plant_Pathogen-Undefined_Saprotroph | 0.21 (0.16) | 1.75 (2.19) | 0.42063 | ns |

**Table S12.** Differences in fungal community function (top 10 in abundance) between FP and CP were determined using nonparametric wilcoxon rank sum test (10 pairs of functional characteristics). CP: Unburned *Pinus massoniana* forest; FP: Burned *Pinus massoniana* forest.

| Comparison | Taxa | FP  (mean ± sd) | CP  (mean ± sd) | *P-values* | Significance |
| --- | --- | --- | --- | --- | --- |
| FP - CP | Ectomycorrhizal | 6.46 (11.17) | 37.41 (28.68) | 0.0317 | * |
| FP - CP | Undefined_Saprotroph | 32.91 (30.98) | 11.48 (12.18) | 0.2222 | ns |
| FP - CP | Ectomycorrhizal-Fungal_Parasite | 0.02 (0.02) | 35.83 (32.84) | 0.0119 | * |
| FP - CP | Ectomycorrhizal-Undefined_Saprotroph | 2.85 (5.46) | 0.32 (0.63) | 0.3457 | ns |
| FP - CP | Undefined_Saprotroph-Undefined_Biotroph | 19.42 (26.98) | 0.01 (0.02) | 0.0212 | * |
| FP - CP | Soil_Saprotroph | 9.81 (13.18) | 0.62 (1.02) | 0.0317 | * |
| FP - CP | Plant_Pathogen | 7.03 (6.90) | 4.53 (8.30) | 0.1508 | ns |
| FP - CP | Wood_Saprotroph | 6.90 (14.07) | 0.97 (1.16) | 0.6905 | ns |
| FP - CP | Animal_Pathogen | 0.68 (0.27) | 3.49 (1.95) | 0.0079 | ** |
| FP - CP | Animal_Pathogen-Plant_Pathogen-Undefined_Saprotroph | 1.97 (3.33) | 0.12 (0.11) | 0.0952 | ns |

**Table S13.** Differences in fungal community function (top 10 in abundance) between FQ and FP were determined using nonparametric wilcoxon rank sum test (10 pairs of functional characteristics). FQ: Burned *Quercus fabri* forest; FP: Burned *Pinus massoniana* forest.

| Comparison | Taxa | FQ  (mean ± sd) | FP  (mean ± sd) | *P-values* | Significance |
| --- | --- | --- | --- | --- | --- |
| FQ - FP | Ectomycorrhizal | 46.09 (21.83) | 6.46 (11.17) | 0.0159 | * |
| FQ - FP | Undefined_Saprotroph | 13.06 (5.48) | 32.91 (30.98) | 0.5476 | ns |
| FQ - FP | Ectomycorrhizal-Fungal_Parasite | 0.18 (0.19) | 0.02 (0.02) | 0.0907 | ns |
| FQ - FP | Ectomycorrhizal-Undefined_Saprotroph | 15.09 (19.32) | 2.85 (5.46) | 0.1508 | ns |
| FQ - FP | Undefined_Saprotroph-Undefined_Biotroph | 0.08 (0.08) | 19.42 (26.98) | 0.2222 | ns |
| FQ - FP | Soil_Saprotroph | 4.20 (8.07) | 9.81 (13.18) | 0.1508 | ns |
| FQ - FP | Plant_Pathogen | 3.25 (1.74) | 7.03 (6.90) | 0.5476 | ns |
| FQ - FP | Wood_Saprotroph | 0.84 (1.35) | 6.90 (14.07) | 0.5476 | ns |
| FQ - FP | Animal_Pathogen | 0.49 (0.23) | 0.68 (0.27) | 0.3095 | ns |
| FQ - FP | Animal_Pathogen-Plant_Pathogen-Undefined_Saprotroph | 0.21 (0.16) | 1.97 (3.33) | 0.42063 | ns |


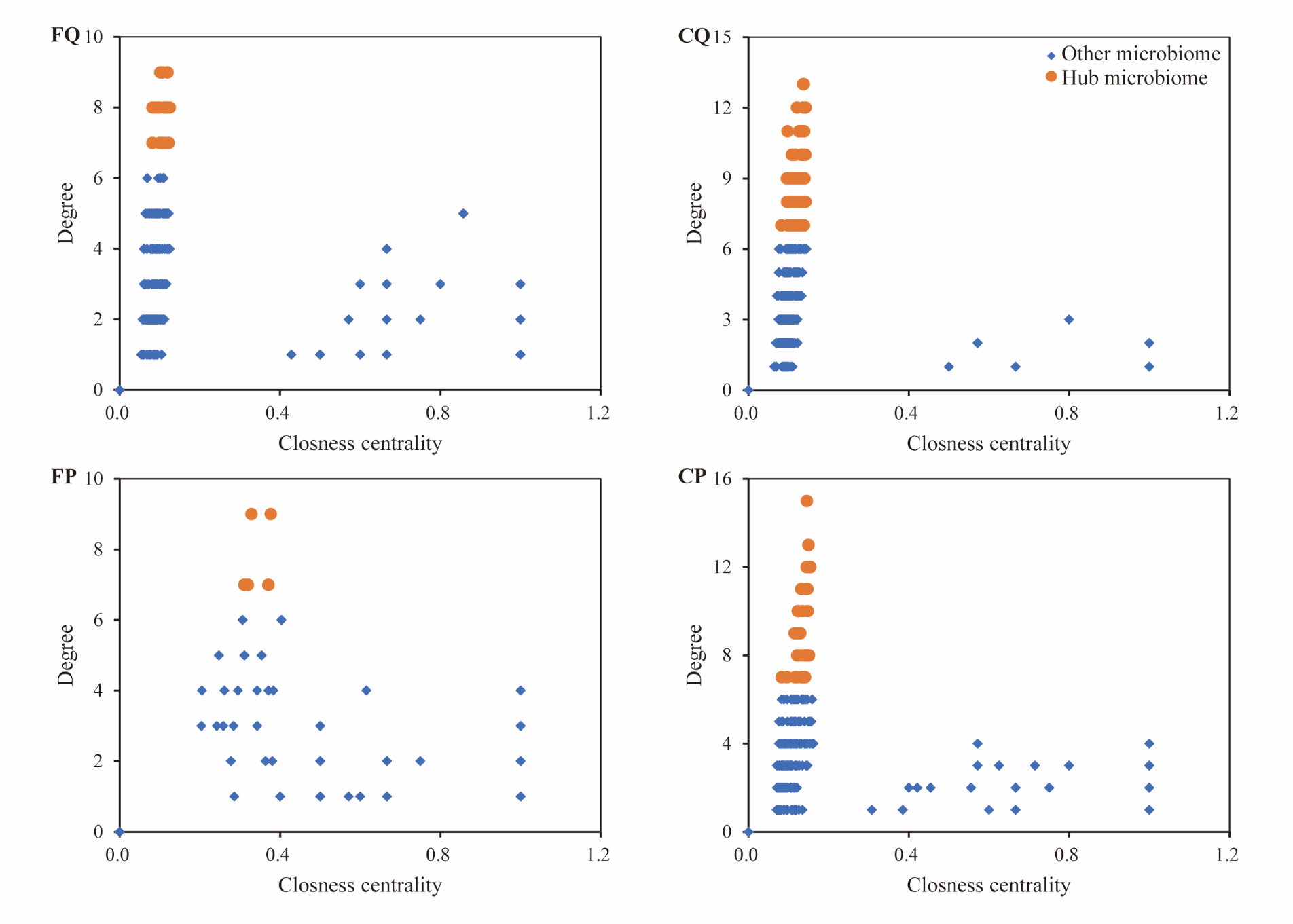


**Figure S1.** Comparison of node level topological features (degree and closeness centrality) of soil microbial network between unburned and burned. CQ: Unburned *Quercus fabri* forest; FQ: Burned *Quercus fabri* forest; CP: Unburned *Pinus massoniana* forest; FP: Burned *Pinus massoniana* forest.

**
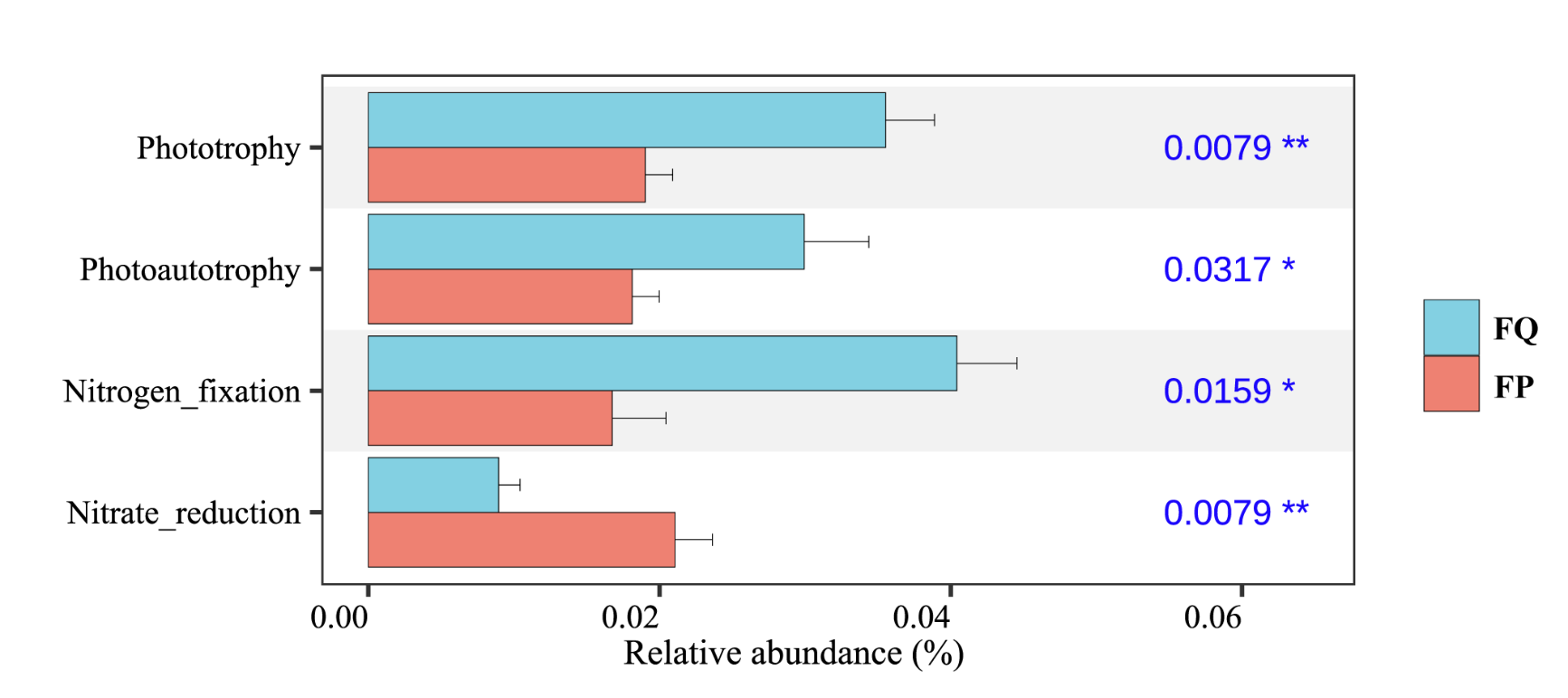
**

**Figure S2.** Differences in bacterial community function (top 10 in abundance) between burned *Quercus fabri* forest and Burned *Pinus massoniana* forest were determined using nonparametric wilcoxon rank sum test. Figure shows only the functional groups with significant (p < 0.05) levels of difference in abundance between burned *Quercus fabri* forest and Burned *Pinus massoniana* forest. Significance levels were *p < 0.05, **p < 0.01. FQ: Burned *Quercus fabri* forest; FP: Burned *Pinus massoniana* forest.

**
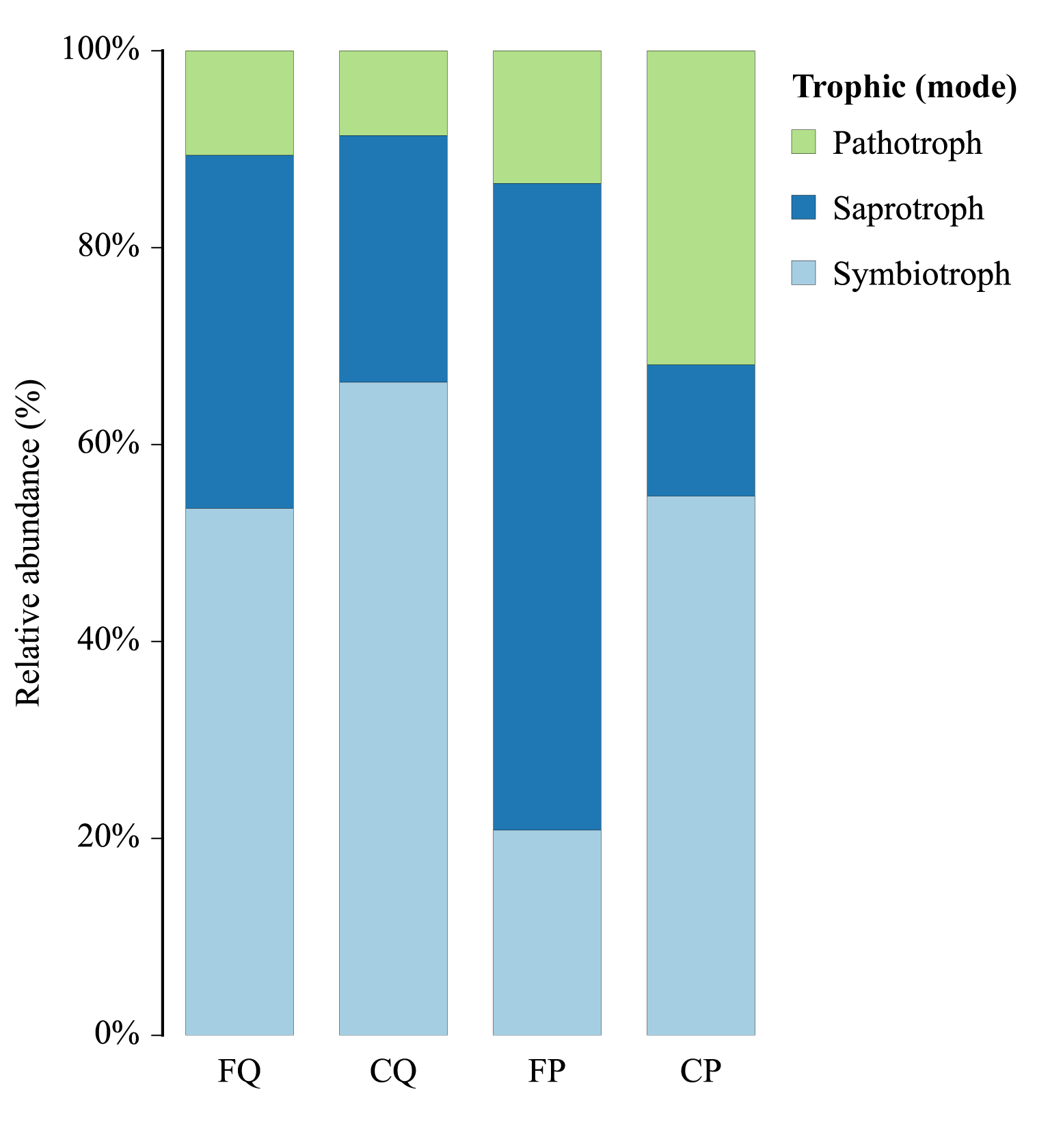
**

**Figure S3.** Functional (top 10 in abundance) composition of fungal community in burned and unburned. CQ: Unburned *Quercus fabri* forest; FQ: Burned *Quercus fabri* forest; CP: Unburned *Pinus massoniana* forest; FP: Burned *Pinus massoniana* forest.

**
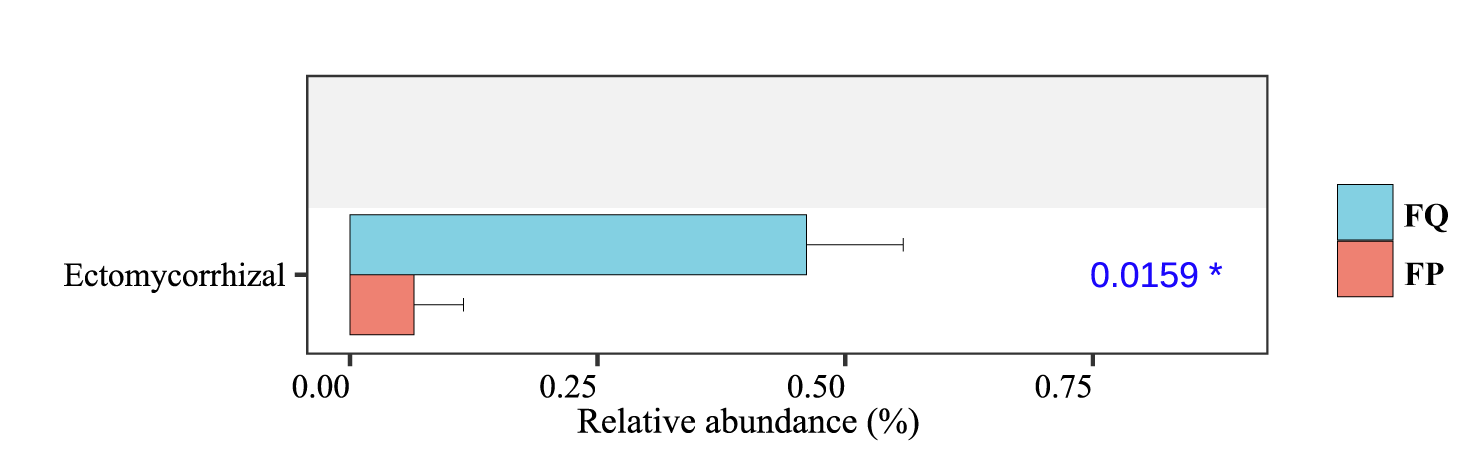
**

**Figure S4.** Differences in fungi community function (top 10 in abundance) between burned *Quercus fabri* forest and Burned *Pinus massoniana* forest were determined using nonparametric wilcoxon rank sum test. Figure shows only the functional groups with significant (p < 0.05) levels of difference in abundance between burned *Quercus fabri* forest and Burned *Pinus massoniana* forest. Significance levels were *p < 0.05, **p < 0.01. FQ: Burned *Quercus fabri* forest; FP: Burned *Pinus massoniana* forest.


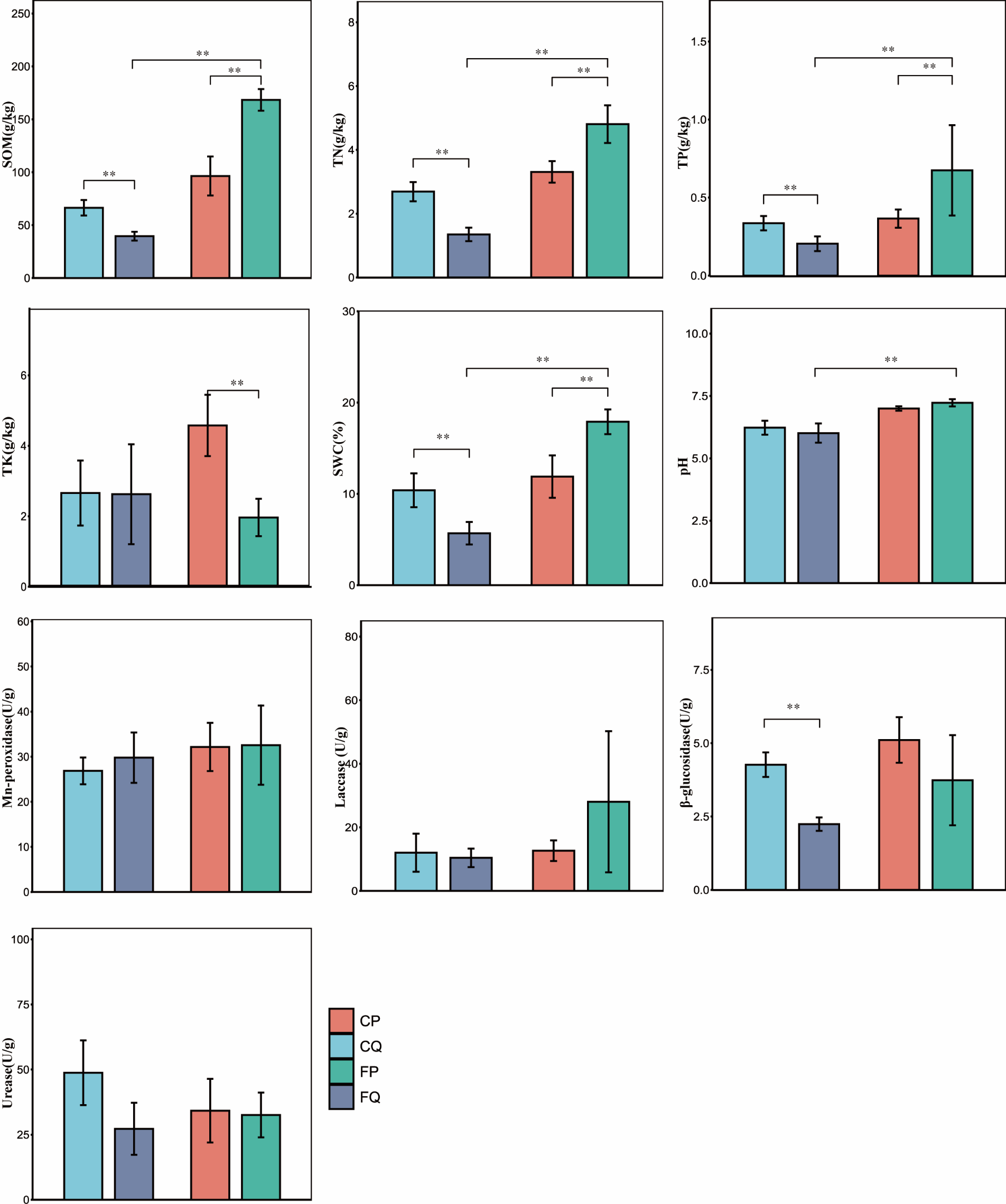


**Figure S5.** The soil properties after fire disturbance. Values are means ± standard error with 15 replicates. The significance of differences sample groups was determined by the nonparametric wilcoxon rank sum test, *Indicates significance, with significance levels of * p < 0.05, and ** p < 0.01. CQ: Unburned *Quercus fabri* forest; FQ: Burned *Quercus fabri* forest; CP: Unburned *Pinus massoniana* forest; FP: Burned *Pinus massoniana* forest. SOM: soil organic matter; TN: total nitrogen; TP: total phosphorus; TK: total potassium; SWC: soil water content.


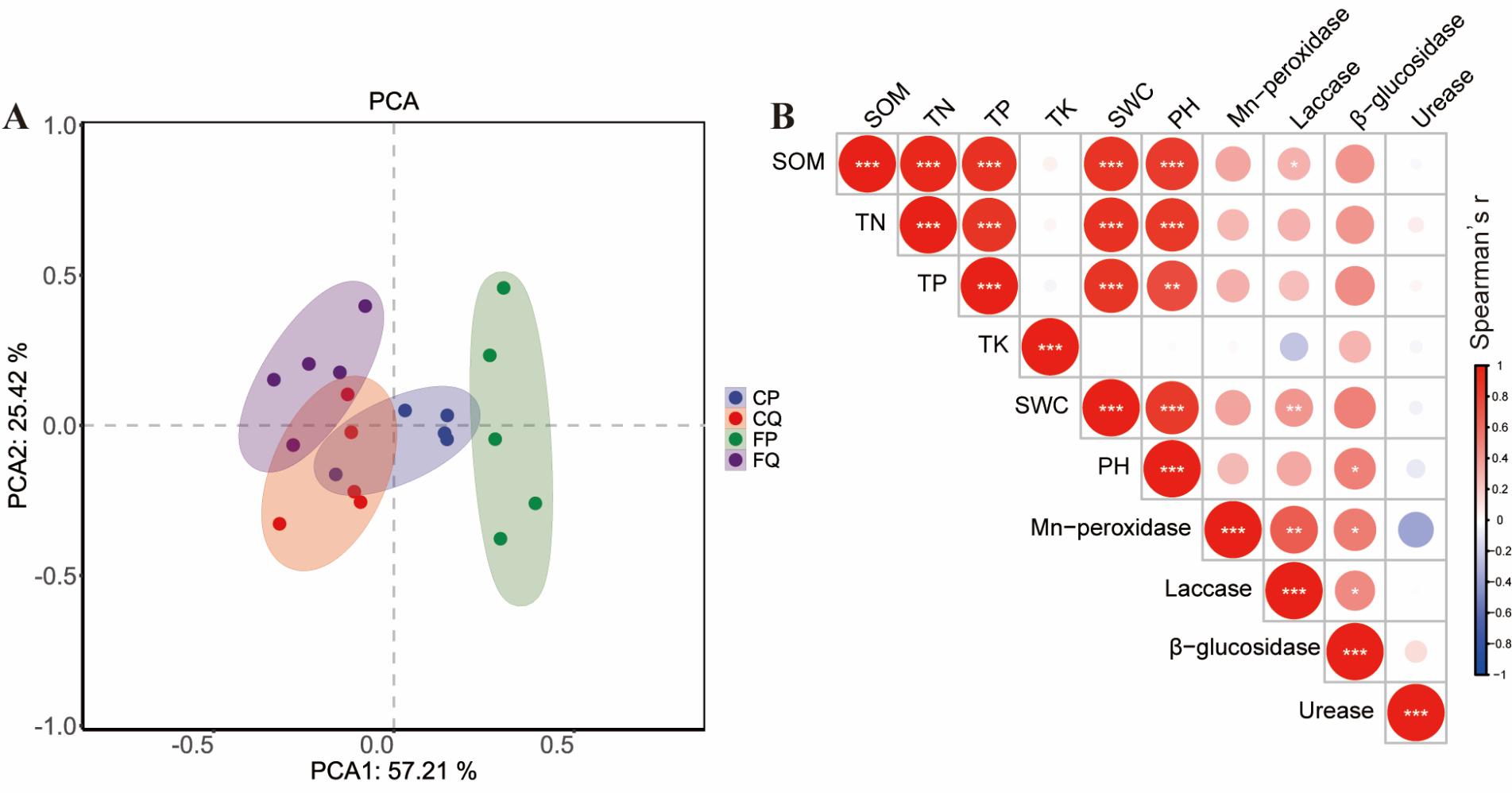


**Figure S6.** (A) Principal component analysis with soil physicochemical factors as variables. (B) Correlation heat map of 10 soil physicochemical factors. *Indicates significance, with significance levels of * p < 0.05, ** p < 0.01, and *** <0.001. SOM: soil organic matter; TN: total nitrogen; TP: total phosphorus; TK: total potassium; SWC: soil water content.
